# Prevalence and natural history of depression after stroke: A systematic review and meta-analysis of observational studies

**DOI:** 10.1371/journal.pmed.1004200

**Published:** 2023-03-28

**Authors:** Lu Liu, Min Xu, Iain J. Marshall, Charles DA Wolfe, Yanzhong Wang, Matthew DL O’Connell

**Affiliations:** 1 School of Life Course and Population Sciences, King’s College London, London, United Kingdom; 2 NIHR Applied Research Collaboration (ARC) South London, London, United Kingdom; 3 National Institute for Health Research (NIHR) Biomedical Research Centre (BRC), Guy’s and St Thomas’ NHS Foundation Trust and King’s College London, London, United Kingdom; Columbia University, UNITED STATES

## Abstract

**Background:**

Depression is the most frequent psychiatric condition after stroke and is associated with negative health outcomes. We aim to undertake a systematic review and meta-analysis of the prevalence and natural history of depression after stroke.

**Methods and findings:**

Studies published up to 4 November 2022 on Medline, Embase, PsycINFO, and Web of Science Core Collection were searched. We included studies of adults with stroke, where depression was assessed at a prespecified time point. Studies excluding people with aphasia and history of depression are excluded. Critical Appraisal Skills Programme(CASP) cohort study tool was used to assess risk of bias. A total of 77 studies were included in the pooled estimates of the prevalence of poststroke depression (PSD). The overall prevalence of depression was 27% (95% CI 25 to 30). Prevalence of depression was 24% (95% CI 21 to 28) by clinical interview and 29% (95% CI 25 to 32) by rating scales. Twenty-four studies with more than one assessment time point reported the natural history of PSD. Among people who were depressed within 3 months of stroke, 53% (95% CI 47 to 59) experienced persistent depression, while 44% (95% CI 38 to 50) recovered. The incidence of later depression (3 to 12 months after stroke) was 9% (95% CI 7 to 12). The cumulative incidence during 1 year after stroke was 38% (95% CI 33 to 43), and the majority (71% (95% CI 65 to 76)) of depression had onset within 3 months after stroke. The main limitation of the present study is that excluding people in source studies with severe impairments may produce imprecise estimates of the prevalence of PSD.

**Conclusions:**

In this study, we observed that stroke survivors with early-onset depression (within 3 months after stroke) are at high risks for remaining depressed and make up two-thirds of the incident cases during 1 year after stroke. This highlights the need for ongoing clinical monitoring of patients depressed shortly after stroke.

**Trial Registration:**

PROSPERO CRD42022314146.

## Abstract 2

Lu Liu and colleagues present an updated systematic review and pooled analysis of depression after stroke.

## Author summary

**Why was this study done?:**

- Depression is common after stroke and depressed patients may be more likely to experience further health problems.
- How soon people become depressed after stroke and how many of them recover is unclear.

**What did the researhers do and find?:**

- We performed an updated review and pooled meta-analysis of depression after stroke including 79 studies.
- The prevalence of poststroke depression by clinical interview was about 24%, which was slightly lower than that by rating scales (29%).
- Depression tends to occur within 3 months after stroke and has a high risk of becoming persistent.

**What do these findings mean?:**

- Those with depression within 3 months after stroke may be likely to experience persistent depression.
- Targeted screening of patients at an early stage poststroke may be an opportunity to improve further health outcomes.

## Background

Depressive disorder is considered the most frequent and burdensome mental health complication after stroke. It is well documented that poststroke depression (PSD) is associated with impaired functional ability, poorer quality of life, and increased mortality [1]. Despite its clear importance after stroke, the prevalence of PSD is reported to be underestimated [2]. The most-quoted studies reporting the prevalence of depression after stroke have used meta-analysis to create large databases. Two highly cited meta-analyses, published about 10 years ago, estimated the prevalence of depression was 30% at any time point following stroke [3,4]. Three more recent meta-analyses on the prevalence of PSD were restricted to specific populations [5–7], while another focused solely on the studies based on clinical interviews [8]. It has been well established that the diagnosis of PSD should be based on a structured mental state examination meeting clinical criteria, such as the Diagnostic and Statistical Manual of Mental Disorders (DSM) or the 10th Revision of the International Classification of Diseases (ICD 10) for depression [9,10]. Previous meta-analyses did not differentiate between the prevalence measured using self-reported depression scales and clinical interview diagnoses.

Routine screening for PSD is recommended by several guidelines [11,12]. However, guidelines differ about the optimal time stroke survivors should be screened for PSD. This is because current studies have used different time points and methods to describe the course of PSD. Shi and colleagues identified 89.9% had no/transient depression and 10.1% had persistent/recurrent depression during 1 year after stroke [13]. Ayis and colleagues described 4 trajectories of depressive symptoms up to 5 years poststroke: no depressive symptoms and remain so over time (15.5%), mild symptoms with a slight increase (49.5%), moderate symptoms with a strong deterioration (28.7%), and severe symptoms with tendency for significant improvement followed by an increase (6.3%) [14]. Stokman-Meiland and colleagues demonstrated that no, nonconsistent, and persistent depressive symptoms were present in 62.9%, 25.2%, and 11.9% of patients within 1 year after stroke, respectively [15]. To our knowledge, there is no meta-analysis until now evaluating the pooled estimates of other measures of natural history (e.g., persistence and recovery) to inform clinical management. Understanding the natural history of depression after stroke has the potential to reduce this high socioeconomic and individual burden. It can help determine the optimal time to screen for PSD, and which patients to target for tailored rehabilitation programs.

The aim of the present systematic review and meta-analysis is to provide updated estimates of the prevalence of depression after stroke, including comparison of the prevalence by diagnostic interview and rating scales. We also aim to synthesize the evidence on the natural history of PSD, in terms of the pooled persistence, recovery, incidence, and cumulative incidence rates.

## Methods

This review was conducted according to PRISMA guidelines. The review protocol was registered on PROSPERO: CRD42022314146.

### Inclusion/Exclusion criteria

Our review was restricted to observational studies of adults (>18 years) with a clinical diagnosis of stroke, where depression was assessed at a prespecified time point. Studies defining depression by clinical interview or on a validated rating scale such as the Hospital Anxiety and Depression Scale (HADS) were included. There were no restrictions on the basis of language, sample size, or duration of follow-up. Studies excluding patients with a history of depression were excluded. Communication impairments are common after stroke, with aphasia affecting approximately one-thirds of patients [16]. It is therefore important to include as many of these people as possible. Yet, communication problems are likely to make the assessment of depression more difficult, or sometimes impossible. We therefore took the following approach. Studies that blanket excluded all stroke survivors with aphasia were not included in this review. Studies that included people with aphasia wherever possible, but excluded some (for example, with severe aphasia preventing administering a questionnaire), were included in our review. Studies were also excluded if they had any of the following restrictions:

1. limited to a specific age or sex group or a specific location of stroke lesion;
2. excessive exclusion criteria that limited the generalizabilty of the results, e.g., the exclusion of multiple comorbid chronic conditions and other neurological diseases;
3. employed convenience sampling or retrospective recruitment;
4. depression reported only as a continuous variable and unable to retrieve a categorical assessment;
5. were intervention studies.

### Search strategy and data extraction

We searched the following digital databases: Medline, Embase, PsycINFO, and Web of Science Core Collection from inception to 4 November 2022. The search strategy is shown in the S1 Search Strategy. Eleven systematic reviews identified were hand-searched for relevant studies [3–8,17–21].

One investigator (LL) first screened all titles and abstracts after removing duplicate papers and excluded clearly irrelevant articles. The remaining studies were read in full against the inclusion and exclusion criteria independently by the two investigators (MX and LL). The disagreements and uncertainties in the process were resolved by discussion with a third reviewer (MOC).

If several articles reported outcomes from the same population, publication with the earliest follow-up were included in the meta-analysis. Clinical interview was preferred over rating scales if studies reported both. If two or more rating scales were used to define depression at the same time point in one paper, authors discussed the results of which assessment to be taken in the meta-analysis.

### Quality of evidence

Study quality was assessed using the Critical Appraisal Skills Programme (CASP) cohort study tool, adapting it for our purpose. We judged potential risk of bias based on 5 items: (1) acceptable recruitment; (2) acceptable stroke assessment; (3) acceptable PSD assessment; (4) length of follow-up; and (5) adequacy of follow-up. The maximum score is 5, which means very high quality, 4 means high quality, 3 means acceptable quality, and 1 and 2 means relatively low quality.

### Statistical methods

We undertook several meta-analyses. First, a meta-analysis was performed to obtain pooled estimates of the prevalence of depression according to the assessment criteria for depression: those using clinical interview for diagnosis and those using rating scales. Another 3 meta-analyses were performed based on (1) time of depression assessment from stroke onset: acute phase (up to 1 month, including date of admission to rehabilitation), medium-term phase (1 to 5 months), long-term phase (6 to 12 months), very long-term phase (more than 1 year after stroke); (2) study settings: population, hospital, or rehabilitation studies; and (3) socioeconomic status: studies in developed countries versus those in developing countries. For studies with follow-up assessments at more than one time point, results from the earliest follow-up were included in the pooled estimates of prevalence of PSD. But we also performed a sensitivity analysis using the latest assessment in each study and data from all time points were presented here. Results from clinical interview were used in the pooled estimates of prevalence of PSD if studies reported the prevalence by both clinical interview and rating scales. But a sensitivity analysis using rating scales was performed and data from all assessment tools were presented. Two sensitivity meta-analyses were also undertaken for studies including (a) both haemorrhagic stroke and ischemic stroke, and (b) people with first-ever stroke.

The second part was to obtain the pooled estimates of measures of natural history within 1 year after stroke. Specifically, the proportion of persistent cases up to 1 year (persistence is defined as depression presented at at least 2 time points and the first onset time point is within 3 months), the frequency of recovery at follow-up among patients with depression soon after stroke (0 to 3 months), the frequency of later incident depression between 3 months and 1 year, the cumulative incidence rates within 1 year after stroke, and the proportion of depression starting within the first 3 months among all depression within 1 year after stroke. For studies reporting proportion of recovered, persistent, and incident cases at more than one time point, results from the longest follow-up were taken to perform pooled estimates. As lost to follow-up of depressed patients at initial assessment is presented in some studies, a sensitivity analysis for studies with complete follow-up at all the prespecified time points was performed. A meta-analysis of the natural history of depression over 1 year after stroke was not conducted because of the small number of available studies.

A random-effects model with DerSimonian and Laird method was used to generate pooled estimates because of the high heterogeneity across studies. Funnel plots were used to evaluate any publication bias. Stata 17.0 was used for data analysis.

## Results

**Fig 1:**
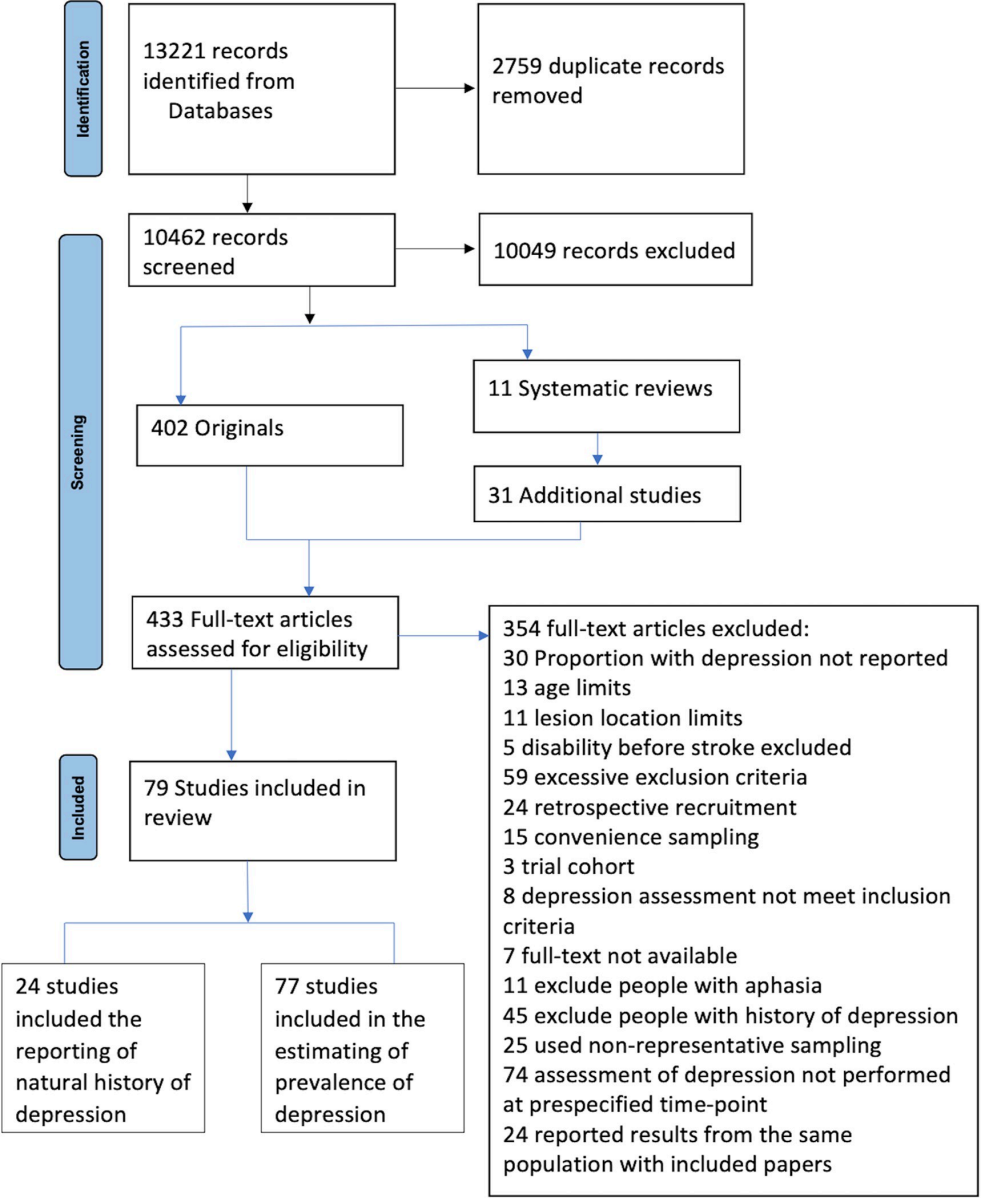
PRISMA flow diagram of literature search.

The initial searches yielded 13,221 references of which 79 met the inclusion criteria. One study only reported the cumulative incidence of PSD during 4 years after stroke and was not included in the pooled estimates [22]. The flow chart is shown in Fig 1.

### Prevalence of depression after stroke

A total of 77 studies [15,23–98] with 27,401 participants were included in the meta-analysis of prevalence of PSD. Twenty-one studies used clinical interview for diagnosis (20 studies [29,31–32,35–37,39,41–42,44,48,62–63,72,83,85–86,90,95,98] using DSM, and 1 study used [59] consensus among clinicians and nurses). The remaining 56 studies assessed depression by rating scales. Different rating scales and differing cutoff choices were used to assess depression. Sixteen different standardized scales were used to assess depression. The cutoff points for the same scale used to assess depression across different studies were not consistent. The most common was the HADS with 3 cutoff points in 17 studies. The Centre for Epidemiologic Studies–Depression Scale (CES-D) was used in 6 studies with 4 cutoff points. The Geriatric Depression Scale (GDS) was used in 6 studies with 4 cutoff points. Five studies identified depression by the Beck Depression Inventory (BDI; 3 cutoff points). Five studies assessed depression with the Patient Health Questionnaire (PHQ; 2 cutoff points). Each of the other 11 rating scales was used in less than 5 studies. Thirty-six studies recruited participants from hospital; 25 studies were population-based studies and 16 were rehabilitation studies. Only 16 studies [30,35,41,44,48,65–66,69,74–75,80–81,84,94–96] assessed depression more than 1 year after stroke. The studies had been undertaken in 28 different countries, of which 69 were in developed countries, while the remaining 8 [24,26,31,36,45–46,49,87] were in low- or middle-income countries (see S1 Table).

**Fig 2:**
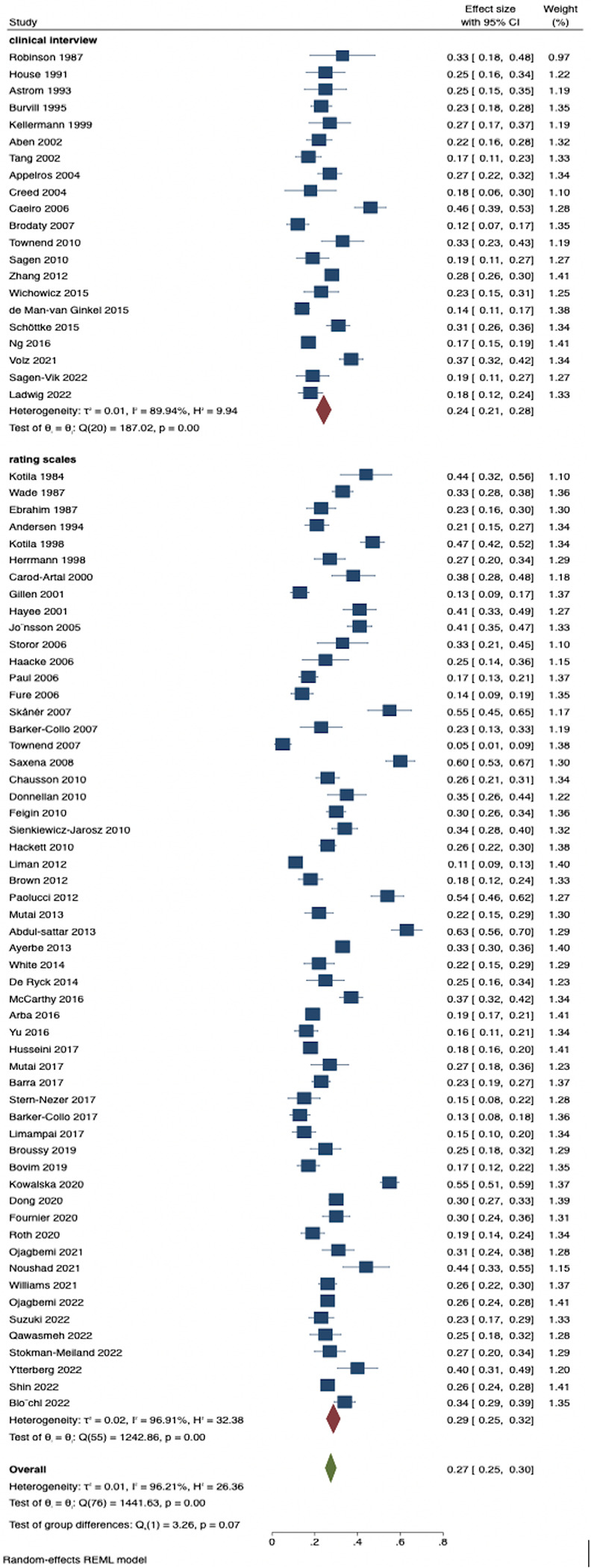
Prevalence of depression stratified by assessment criteria.

The overall pooled estimates for prevalence of PSD at any time point was 27% (95% CI 25 to 30), with a prevalence of 30% (95% CI 24 to 36) within a month of stroke, 27% (95% CI 24 to 30) at 1 to 5 months, 22% (95% CI 18 to 26) at 6 months to 1 year, and 29% (95% CI 22 to 35) at more than 1 year. Pooled prevalence of depression was 24% (95% CI 21 to 28) by clinical interview and 29% (95% CI 25 to 32) by rating scales (results are shown in Fig 2). The prevalence was not significantly different across study settings with a pooled estimate of 25% (95% CI 22 to 28) in hospital studies, 28% (95% CI 24 to 33) in population studies, and 31% (95% CI 23 to 39) in rehabilitation studies. Similarly, the pooled prevalence of depression in studies undertaken in developed countries was 27% (95% CI 25 to 30) and in studies in developing counties was 28% (95% CI 21 to 35). Heterogeneity was significant due to different time points of assessment, differences in assessment method, and heterogeneity of study populations. This overall pooled prevalence rates did not change significantly if the latest assessment time point was used. Results also showed little variation when rating scales were preferred over clinical interview. Results are shown in S1–S5 Figs.

There was no great difference in pooled prevalence of depression across method of case selection. The pooled estimates for the 62 studies that included people with both ischemic stroke and haemorrhagic stroke was 29% (95% CI 26 to 32); for the 24 studies that included people with first-ever stroke, pooled prevalence of depression was 28% (95% CI 24 to 33). Results are shown in S6 and S7 Figs.

### Natural history of depression after stroke

Twenty-four studies with more than one assessment time point reported the natural history of PSD [15,23–44,99]. Eighteen studies [15,23–28,30,32–34,36,38–39,41–44] reported the rates of persistent depression within 1 year. Proportion of recovery from depression within 1 year among patients who are depressed within 3 months after stroke was reported in 16 studies [15,23–28,30,33–34,36,38–39,41–42,44]. Proportion of incident cases between 3 months and 1 year was reported in 16 studies [15,23,25–27,29–33,37,40,42–44,99]. Cumulative incidence rates and proportion of early-onset cases of depression among all patients depressed within 1 year were reported in 16 studies [15,23,25–27,29–33,37,40,42–44,99]. Analysis from participants who completed all the assessments was demonstrated in 9 studies [15,23,25–27,30,33,42,44] (see S2 Table).

**Fig 3:**
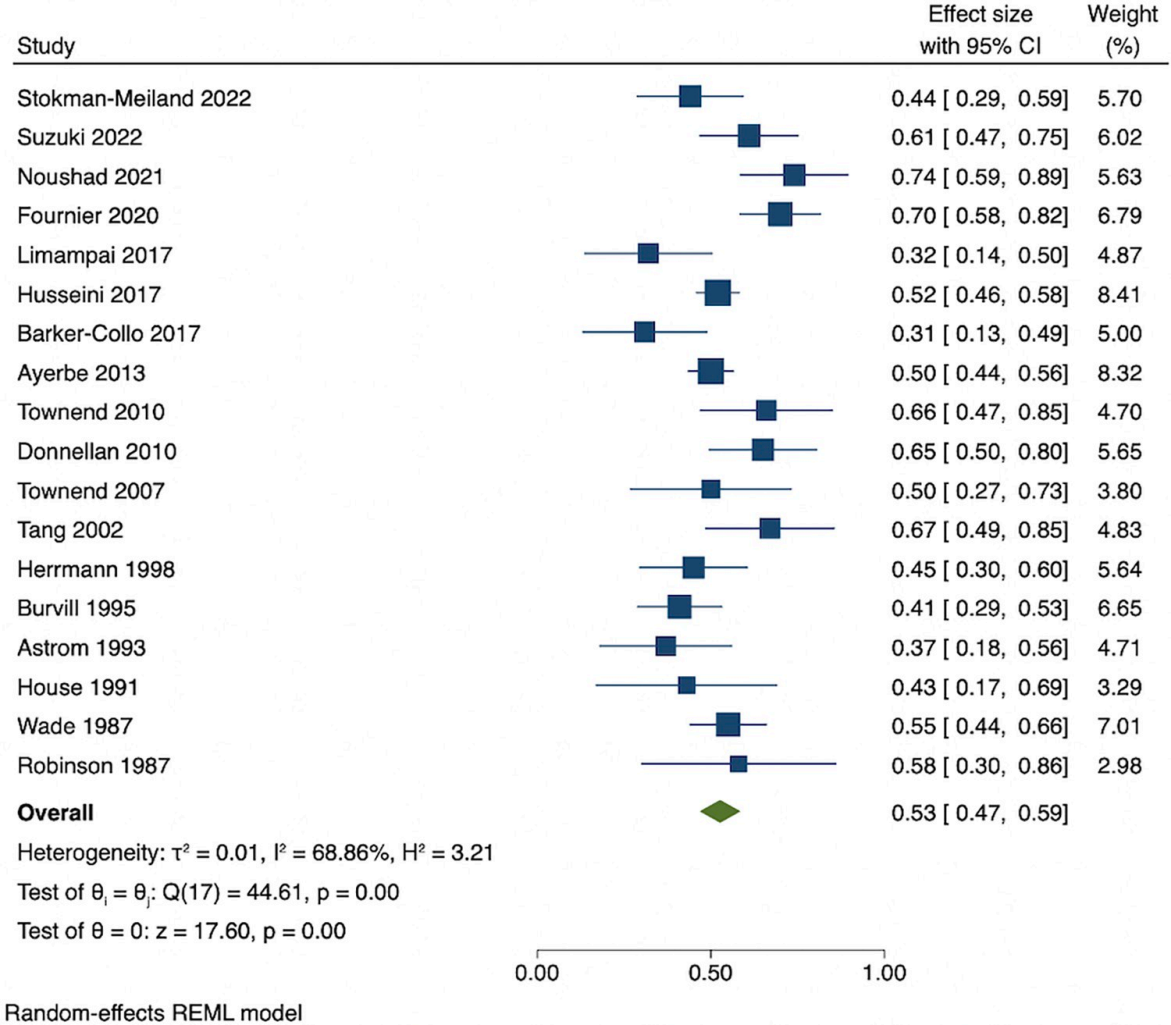
Proportion of persistent depression within 1 year among people who are depressed within 3 months.

**Fig 4:**
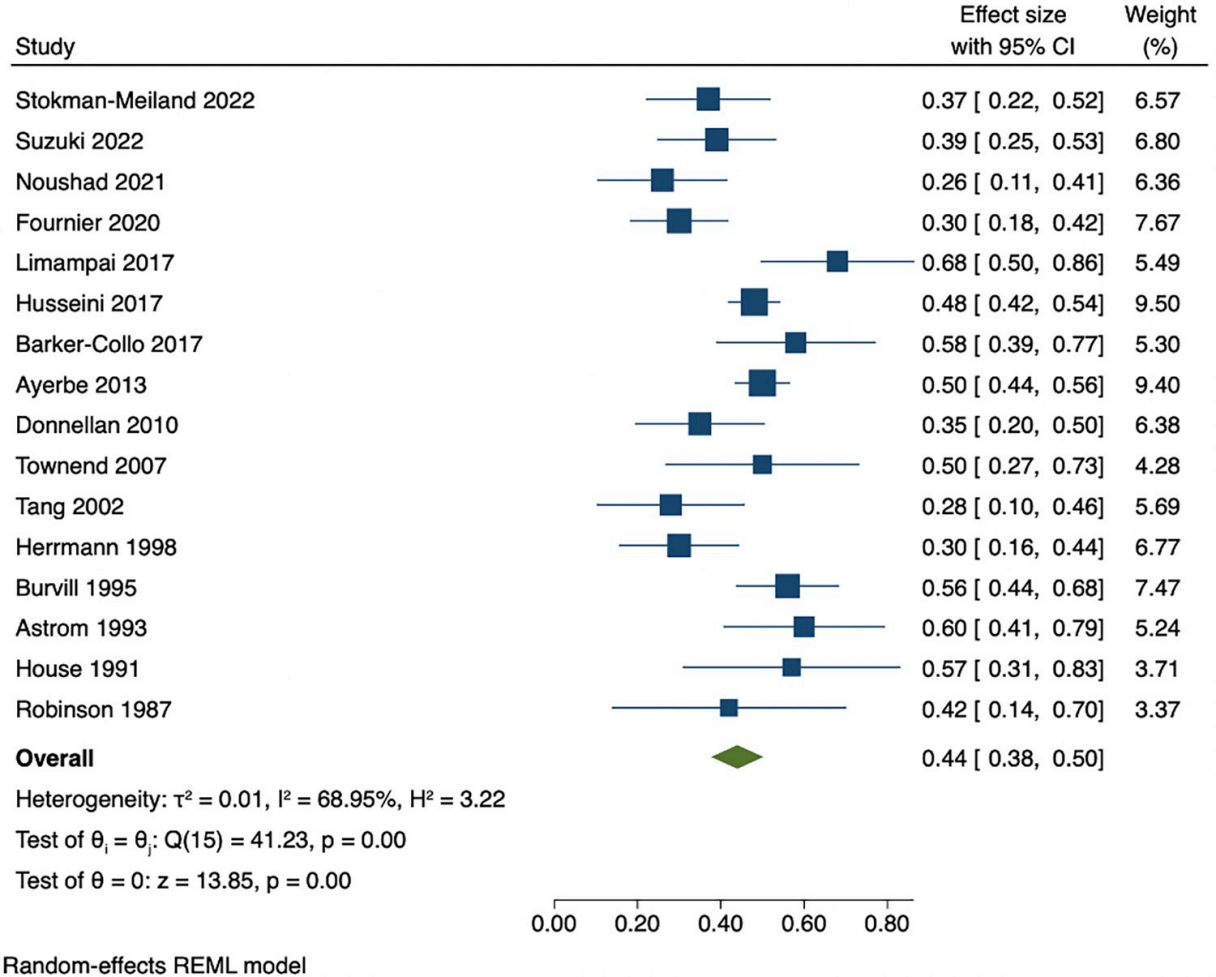
Proportion of recovered patients within 1 year among people who are depressed within 3 months after stroke.

**Fig 5:**
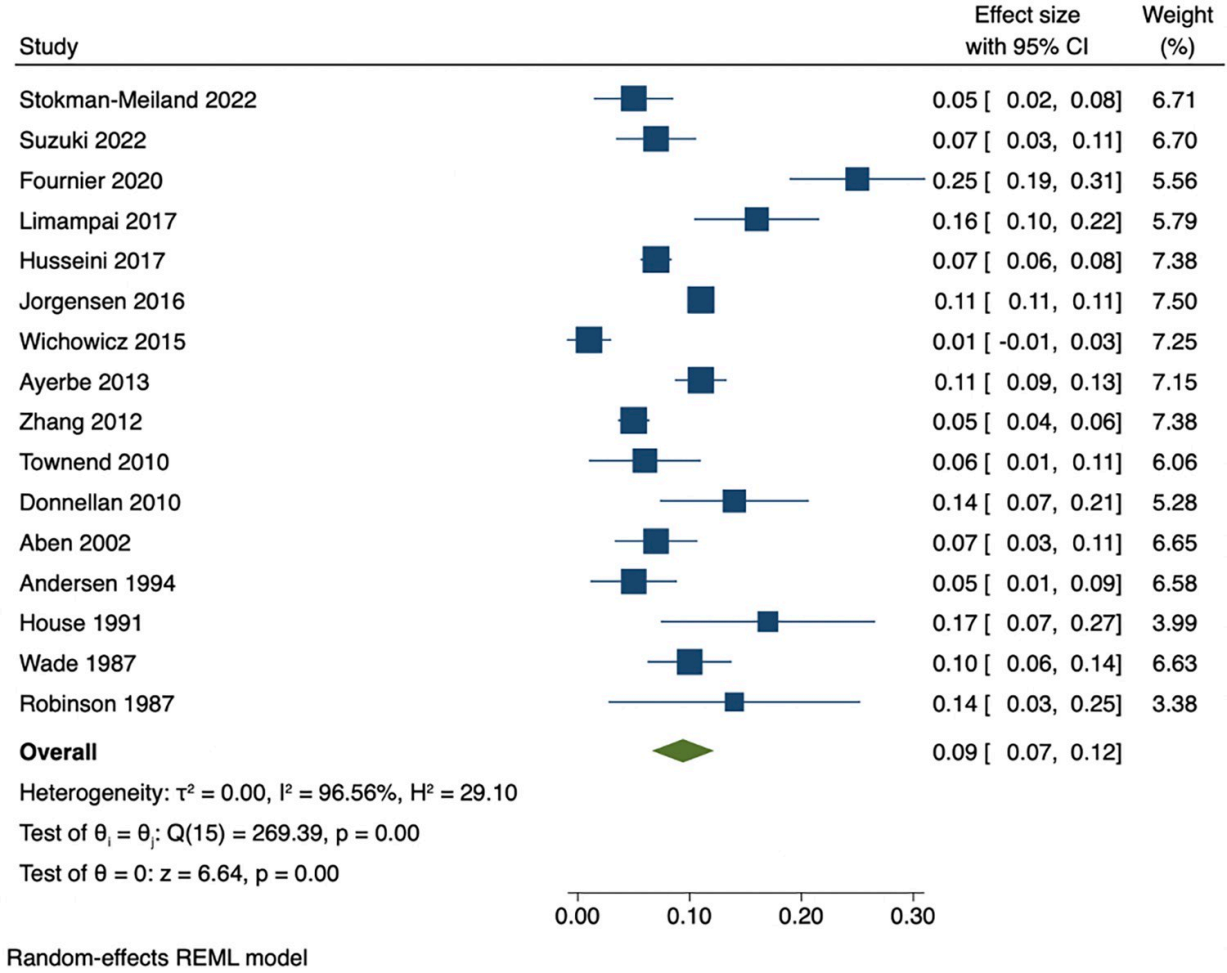
Proportion of incident cases of depression between 3 months and 1 year after stroke.

**Fig 6:**
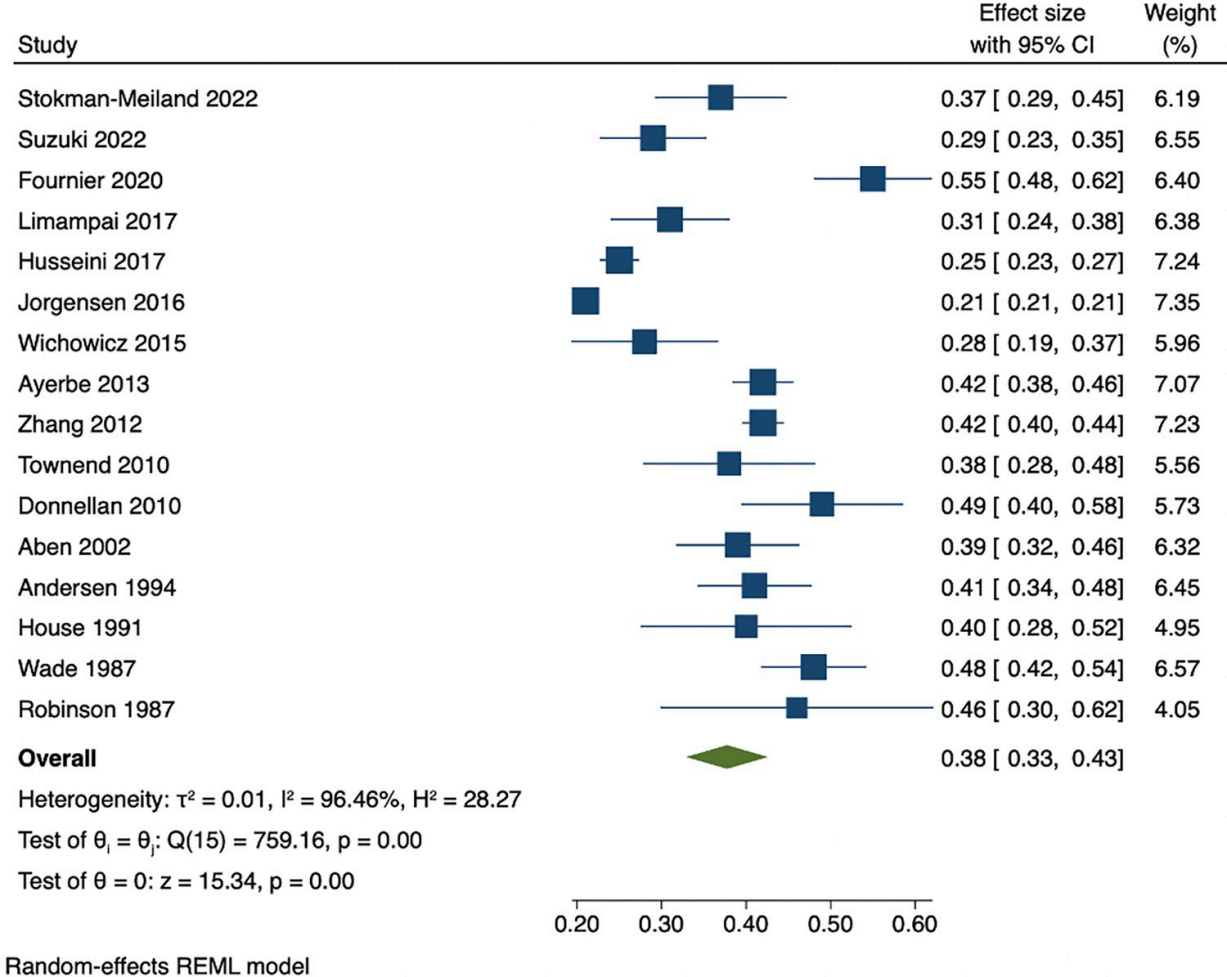
Cumulative incidence of depression within 1 year after stroke.

**Fig 7:**
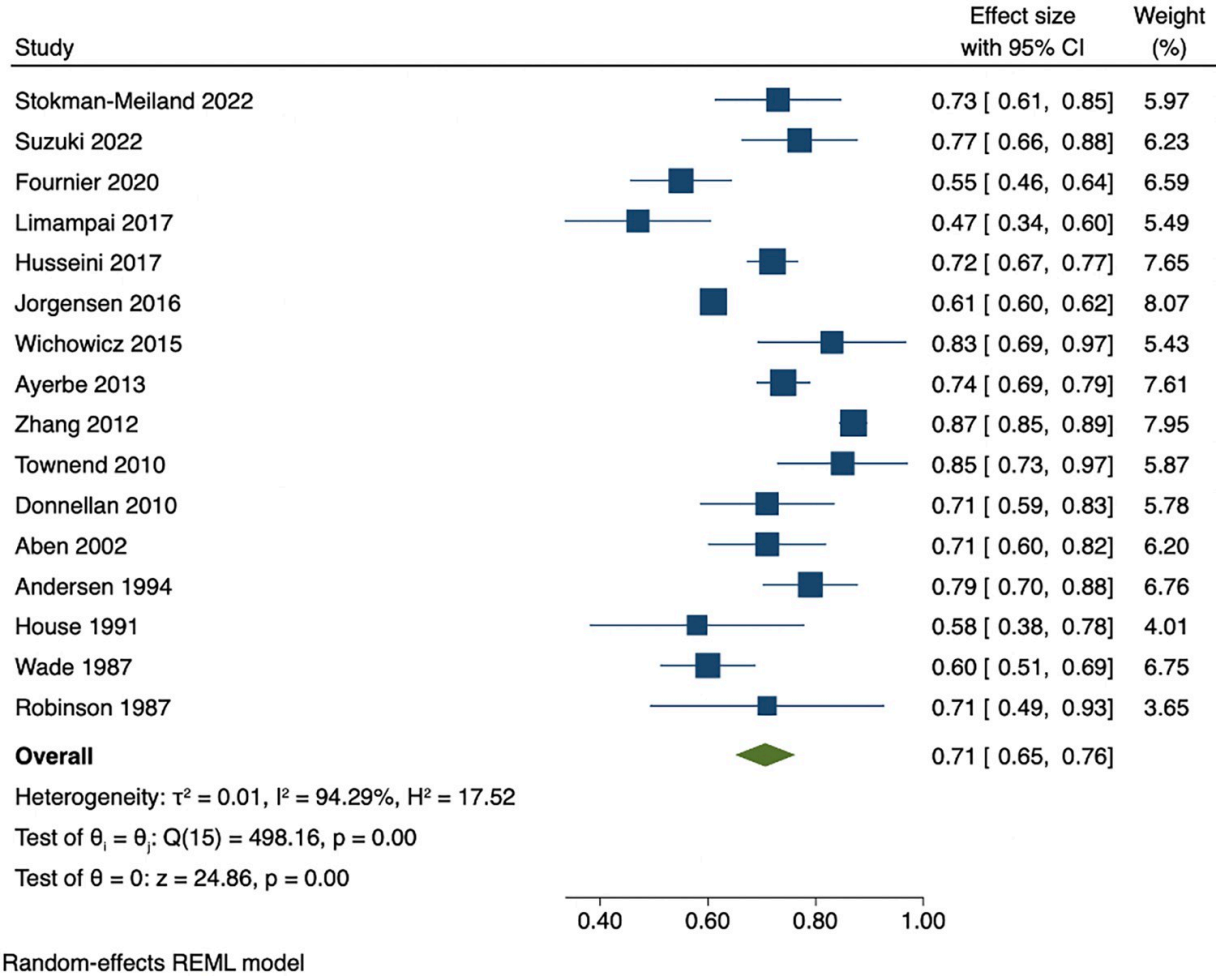
Proportion of early-onset depression (within 3 months after stroke) among all incident cases during 1 year after stroke.

Among people who were depressed within 3 months of stroke, the pooled estimates of persistence within 1 year was 53% (95% CI 47 to 59), while pooled rates of recovery within 1 year was 44% (95% CI 38 to 50). The pooled results showed that proportion of incident cases between 3 months and 1 year after stroke was 9% (95% CI 7 to 12). Cumulative incidence within 1 year ranged from 21% to 55%. The pooled cumulative incidence estimate was 38% (95% CI 33 to 43), and the majority (71% (95% CI 65 to 76)) of cases of depression had onset during the first 3 months after stroke. Results are presented in Figs 3–7. There were no significant differences in measures of natural history of depression following sensitivity analysis for 9 studies in patients with complete assessments at all the time points (see S8–S12 Figs).

Five studies reported natural history of depression over 1 year after stroke. Ayerbe and colleagues reported the poststroke incidence of PSD ranged between 7% and 21% per year during the 15-year follow-up. Cumulative incidence of depression was 55.4% [30]. Astrom and colleagues demonstrated that 36% patients had a long-lasting depression up to 3 years after stroke [41]. Brodatory and colleagues showed that nearly 40% of depressed patients at 3 months after stroke had recovered at 15 months after stroke, while 60% of them experienced persistent depression [35]. In a large sample size study with 135,417 patients with stroke, cumulative incidence rate within 2 years after stroke was 25.4% and more than half of the cases appeared within the first 3 months [99]. Robinson and colleagues found that of patients who were depressed in hospital, 37% had no symptom of depression at 2 years follow-up and the remaining 63% continued to have depression at 2 years after stroke [44].

### Quality assessment and publication bias

Among the 78 studies, low risk of bias was assigned to scale items ranging from 2 out of 5 to 5 out of 5 items. All 78 studies had low risk for acceptable stroke assessment and enough length of follow-up for depression occur. Low risk of bias was presented on acceptable recruitment for 26 studies. Fifty-seven studies showed low risk of bias on adequacy of follow-up. Studies using clinical interview had low risk of bias. Out of 22 studies using clinical interview, 21 scored 4 or 5, while 12 out of 56 studies using rating scales were very high quality or high quality. In total, 33 studies were of very high quality or high quality and 35 studies were acceptable quality. Funnel plot for the studies included is slightly asymmetric, indicating modest publication bias of studies. Specifically, the shape of the plot suggests that smaller studies with low prevalence estimates were less likely to get published (see S3 Table and S13 Fig).

## Discussion

### Summary of the finding

To the best of our knowledge, this is the first study comparing the prevalence of PSD in terms of robust clinical interview versus rating questionnaires. We found that across all studies to date, the pooled estimate of the prevalence of PSD was 27%. This was slightly higher when assessed by rating scales at 29%, compared to 24% diagnosed by clinical interview. Moreover, to our knowledge, this is also the first meta-analysis evaluating the pooled proportion of recovered, persistent, and new-onset cases of depression. The natural history of depression after stroke within 1 year is dynamic. Two-thirds of episodes of depression during 1 year after stroke started within 3 months. At further follow-up, over half of these patients experienced persistent depression and just under half had recovered. Onset of depression was less common after 3 months.

Our results suggested the pooled prevalence of PSD is 27% (95% CI 25 to 30) at any time point after stroke. This is consistent with previous meta-analyses, suggesting the prevalence of PSD has not changed appreciably in more recent studies. This consistency in estimates is notable as earlier studies also varied in whether they included studies excluding participants with prestroke depression. The one meta-analysis to directly address this issue also showed little variation when comparing pooled estimates of depression in studies including people with a history of depression and that in studies excluding people with a history of depression [4]. Studies have shown conflicting results on whether prestroke depression increases the odds of PSD [62,64,100]. The present pooled prevalence in studies including people with aphasia is comparable to that in studies excluding people with aphasia [4]. This may be because most of the studies only included people with mild to moderate aphasia as meaningful communication skills are essential to complete the questionnaire. It remains challenging to estimate the prevalence of PSD in the full stroke population.

Rating scales are widely used to screen for depression as they have the advantage of requiring fewer resources and being easily applicable [101]. These assessment tools have been shown to have a high sensitivity but low specificity [102–104]. The relatively small difference between the two methods in our study suggests only modest overreporting of depression by screening questionnaires [3]. This may be due to patients who are capable to complete the scales are fully self-aware to provide an accurate self-assessment, which might not reflect the real condition. For example, sleep alterations, appetite, and attention deficits might lead patients to overestimate the prevalence of depression [105].

In the sensitivity analyses, the pooled estimates of depression when restricted to studies that included both ischemic stroke and haemorrhagic stroke patients were similar to the overall pooled estimate. Comparison of PSD between ischemic and haemorrhagic stroke patients was less frequently demonstrated, and results were conflicting. Zeng and colleagues reported nearly twice the prevalence of PSD in intracerebral haemorrhage group than that in ischemic stroke group [106], while the prevalence of depression after ischemic stroke and haemorrhagic stroke was similar in Sienkiewicz-Jarosz’s study [71]. The experience of depression may vary across socioeconomic and clinical circumstances. However, no significant difference was seen between developed countries and low- or middle-income countries. It may result from unrecognised depression in developing countries. As seen in our results, only 8 studies were in low- or middle-income countries.

Pooled estimates of studies assessing patients at more than one time point showed that about half of patients with depression soon after stroke experienced persistent depression and that the remaining 50% patients recovered from this problem within 1 year. The frequency of new-onset depression between 3 and 12 months after stroke was 9% (95% CI 7 to 12). The systematic review by Ayerbe and colleagues [3] found that PSD presented a dynamic natural history, with new cases and recovery of depression occurring over time. Here, we confirmed and extended their findings by providing formal meta-analysis and pooled estimates of measures of natural history of depression after stroke from the larger number of studies now available. Our study also found two-thirds of PSD onset within 1 year occur during the first 3 months. It indicates that early-onset depression makes up the majority of episodes during 1 year after stroke, while onset of depression was less common after 3 months.

A possible explanation for the dynamic nature of PSD is the level of concurrent functional deficits experienced. Studies comparing patients with persistent depression and nonpersistent depression found that absolute degree of functional disability and poorer health status over time are associated with persistent and progressive symptoms. Correspondingly, as functional and intellectual performance improve, depressive symptoms decreased [25,27,31,43]. Another study found that stroke survivors with persistent depression were those with a prestroke history of psychiatric problems [42]. Based on these results, it is recommended that clinical attention should be paid to patients with depressive symptoms within 3 months poststroke, especially those with worse functional impairments at baseline and those with prestroke depression. Finally, about 10% of new cases of depression had onset between 3 months and 1 year after stroke, which comprised 30% of the cumulative cases within 1 year. Whether patients with late-onset depression have a high risk of experiencing persistent depression is unknown since there are insufficient data to estimate the natural history of PSD over 1 year. Population-based studies investigating the prevalence, incidence of PSD at different time points, and the persistent patterns with long-term follow-up are needed.

### Source of heterogeneity

Heterogeneity was significant in the present study, which is similar to previous meta-analyses evaluating the prevalence of PSD. Differing times of symptom assessment, differences in diagnostic criteria, heterogeneity of sociodemographic characteristics of patients enrolled, and differing choices of cutoff points that define depression were factors that contributed to differences in these estimates. We explored 4 sources of heterogeneity a priori: method of depression assessment, study setting, times of symptom assessment, and socioeconomic characteristics. When stratified by depression assessment, the prevalence of depression by rating scale is a slightly lower than that by clinical interview. There is no difference in the prevalence of PSD when classified by study setting. Times of depression assessment did not significantly influence the prevalence estimate, nor did the socioeconomic characteristics.

### Strength and limitations

There are some limitations of our study. First, most of the source studies excluding people with severe impairments (e.g., severe aphasia) may produce imprecise estimates of the prevalence of PSD. Second, the number of studies included in estimating the pooled results of natural history is not large enough to stratify the studies by the use of clinical interview or rating scales. Subgroup analysis in studies with population, hospital, and rehabilitation settings is also unavailable. Third, we did not search unpublished studies. Whether these studies would potentially change the results is unknown. Fourth, the prevalence estimates obtained from our meta-analysis were calculated for all combinations of standardized scales and thresholds; there is inadequate data to do subgroup analysis in studies using same thresholds on same rating scales. These measurement differences are one source of heterogeneity across studies. Future studies could explore a more formal analysis of this issue. For example, using a Bayesian framework to get all measures onto a common scale and defining comparable thresholds for the different rating scales based on population norms. The strength of the present meta-analysis is the inclusion of studies including people with aphasia and history of depression, increasing the generalizability. All the systematic reviews identified were hand-searched for relevant studies, which decreased the number of missed studies. The large number of included studies enabled us to undertake more subgroup analyses than previous studies.

### Clinical and research implication

Patients with early-onset (within 3 months after stroke) depression have a high risk of experiencing persistent depression, especially for patients with worse baseline functional disability and those with prestroke depression. Targeted monitoring and tailored intervention may improve outcomes. Appropriate interventions for depression, such as a combination of pharmacological intervention and psychological therapy, may enhance the stroke rehabilitation. Late-onset (between 3 months and 1 year after stroke) depression is less common within 1 year after stroke. But the natural history of the late-onset depression is unknown. Population-based studies investigating the prevalence, incidence of PSD at different time points, and persistent patterns with long-term follow-up are needed. Second, people with aphasia are frequently excluded from depression screening due to inability to participate. It remains challenging to evaluate the accurate prevalence and incidence of PSD. Representative studies, including patients with communication or cognitive impairments, are needed to provide generalisable estimates of the natural history of depression poststroke. Finally, depression is less frequently reported in low- or middle-income countries. More attention should be paid to psychological disorders after stroke in low- and middle-income counties since more than two-thirds of the worldwide burden of stroke comes from these countries [107].

## Conclusions

Our findings indicate that rating scales may slightly overreport the prevalence of depression after stroke, compared to clinical interview. These scales possibly capture a proportion of patients with milder symptoms than would be confirmed as depressed at clinical interview. More importantly, early-onset depression after stroke requires periodic clinical attention in the long term as it has a high risk of becoming persistent depression, especially for patients with severe baseline functional disability and history of depression. The early identification of PSD would help clinicians to make tailored rehabilitation and prevention strategies to the affected patients. Finally, in order to understand the long-term course of PSD, studies demonstrating incidence and prevalence of depression beyond 1 year after stroke, the time of depression onset, and patterns of recovery and recurrence are needed.

## Supporting information

S1 Search Strategy Search strategy. (DOCX)

S1 Protocol Registry Protocol registry details. (DOCX)

S1 PRISMA Checklist PRISMA 2020 checklist. (DOCX)

S1 Table Prevalence of depression after stroke. (DOCX)

S2 Table Natural history of poststroke depression. (DOCX)

S3 Table Risk of bias assessment. (DOCX)

S1 Fig Prevalence of depression stratified by assessment time points. (PDF)

S2 Fig Prevalence of depression stratified by study setting. (PDF)

S3 Fig Prevalence of depression in terms of socioeconomic status. (PDF)

S4 Fig Prevalence of depression when using data from latest assessment. (PDF)

S5 Fig Prevalence of depression after stroke when rating scales are preferred over clinical interview. (PDF)

S6 Fig Prevalence of depression in studies with both ischemic stroke and haemorrhagic stroke. (PDF)

S7 Fig Prevalence of depression in studies with first-ever stroke. (PDF)

S8 Fig Proportion of recovered patients within 1 year among people who are depressed within 3 months after stroke for studies with analysis in participants completed follow-up at all prespecified time points. (PDF)

S9 Fig Proportion of persistent depression within 1 year among people who are depressed within 3 months after stroke for studies with analysis in participants completed follow-up at all prespecified time points. (PDF)

S10 Fig Proportion of incident cases between 3 months and 1 year after stroke for studies with analysis in participants completed follow-up at all prespecified time points. (PDF)

S11 Fig Cumulative incidence of depression within 1 year after stroke for studies with analysis in participants completed follow-up at all prespecified time points. (PDF)

S12 Fig Proportion of early-onset depression (within 3 months after stroke) in all incident cases within 1 year for studies with analysis of participants completed follow-up at all prespecified time points. (PDF)

S13 Fig Funnel plot for the studies included. (TIF)

## Abbreviations

BDI: Beck Depression Inventory
CASP: Critical Appraisal Skills Programme
CES-D: Centre for Epidemiologic Studies–Depression Scale
DSM: Diagnostic and Statistical Manual of Mental Disorders
GDS: Geriatric Depression Scale
HADS: Hospital Anxiety and Depression Scale
ICD 10: 10th Revision of the International Classification of Diseases
PHQ: Patient Health Questionnaire
PSD: poststroke depression
