# Supplementary material for: Prevalence and natural history of depression after stroke: A systematic review and meta-analysis of observational studies
Source: PLoS Med. 2023 Mar 28;20(3):e1004200. doi: 10.1371/journal.pmed.1004200 (PMC10047522; doi:10.1371/journal.pmed.1004200)
Supplement: S1 Search Strategy — (DOCX) [file pmed.1004200.s001.docx]

**S1 Search Strategy**

**Ovid (Medline, Embase, PsycINFO)**

1. stroke*.mp. [mp=ti, ot, ab, nm, hw, ui, an, tc, id, sh, tn, dm, mf]

2. poststroke*.mp. [mp=ti, ot, ab, nm, hw, ui, an, tc, id, sh, tn, dm, mf]

3. post-stroke*.mp. [mp=ti, ot, ab, nm, hw, ui, an, tc, id, sh, tn, dm, mf]

4.1 or 2 or 3

5. infarct*.mp. [mp=ti, ot, ab, nm, hw, ui, an, tc, id, sh, tn, dm, mf]

6. isch?emi*.mp. [mp=ti, ot, ab, nm, hw, ui, an, tc, id, sh, tn, dm, mf]

7. thrombo*.mp. [mp=ti, ot, ab, nm, hw, ui, an, tc, id, sh, tn, dm, mf]

8. emboli*.mp. [mp=ti, ot, ab, nm, hw, ui, an, tc, id, sh, tn, dm, mf]

9. apoplexy.mp. [mp=ti, ot, ab, nm, hw, ui, an, tc, id, sh, tn, dm, mf]

10. 5 or 6 or 7 or 8 or 9

11. cerebral.mp. [mp=ti, ot, ab, nm, hw, ui, an, tc, id, sh, tn, dm, mf]

12. brain*.mp. [mp=ti, ot, ab, nm, hw, ui, an, tc, id, sh, tn, dm, mf]

13. cerebellar.mp. [mp=ti, ot, ab, nm, hw, ui, an, tc, id, sh, tn, dm, mf]

14. cerebellum*.mp. [mp=ti, ot, ab, nm, hw, ui, an, tc, id, sh, tn, dm, mf]

15. vertebrobasilar.mp. [mp=ti, ot, ab, nm, hw, ui, an, tc, id, sh, tn, dm, mf]

16. intracerebral.mp. [mp=ti, ot, ab, nm, hw, ui, an, tc, id, sh, tn, dm, mf]

17. intracranial.mp. [mp=ti, ot, ab, nm, hw, ui, an, tc, id, sh, tn, dm, mf]

18. subarachnoid.mp. [mp=ti, ot, ab, nm, hw, ui, an, tc, id, sh, tn, dm, mf]

19. 11 or 12 or 13 or 14 or 15 or 16 or 17

20.11 or 12 or 13 or 14 or 15 or 16 or 17 or 18

21. h?emorrhage.mp. [mp=ti, ot, ab, nm, hw, ui, an, tc, id, sh, tn, dm, mf]

22. bleed.mp. [mp=ti, ot, ab, nm, hw, ui, an, tc, id, sh, tn, dm, mf]

23. 21 or 22

24. 10 and 19

25. 20 and 23

26. 4 or 24 or 25

27. depressi*.mp. [mp=ti, ot, ab, nm, hw, ui, an, tc, id, sh, tn, dm, mf]

28. depressive disorder*.mp. [mp=ti, ot, ab, nm, hw, ui, an, tc, id, sh, tn, dm, mf]

29. exp depression/

30. exp depressive disorder/

31. 27 or 28 or 29 or 30

32. frequenc*.mp. [mp=ti, ot, ab, nm, hw, ui, an, tc, id, sh, tn, dm, mf]

33. incidence*.mp. [mp=ti, ot, ab, nm, hw, ui, an, tc, id, sh, tn, dm, mf]

34. prevalence*.mp. [mp=ti, ot, ab, nm, hw, ui, an, tc, id, sh, tn, dm, mf]

35. "natural histor*".mp. [mp=ti, ot, ab, nm, hw, ui, an, tc, id, sh, tn, dm, mf]

36. 32 or 33 or 34 or 35

37. 26 and 31 and 36

38. limit 37 to humans

**Web of Science Core Collection**=SCI-EXPANDED, SSCI, A&HCI

#1. AF =("cerebral infarct*"OR "cerebral isch?emi*"OR "cerebral thrombo*" OR "cerebral emboli*"OR "cerebral apoplexy" OR "brain*infarct*"OR"brain* isch?emi*"OR"brain*thrombo*"OR "brain*emboli*"OR "brain* apoplexy"OR "cerebral h?emorrhage"OR "cerebral bleed" OR "brain* h?emorrhage" OR "brain* bleed"OR "intracerebral h?emorrhage" OR "intracerebral bleed" OR "intracranial h?emorrhage" OR "intracranial bleed" OR "subarachnoid h?emorrhage" OR "subarachnoid bleed" OR stroke* OR poststroke* OR post-stroke*)

#2. AF= (depressi* OR “depressive disorder*”)

#3. AF= (frequenc* OR incidence* OR prevalence* OR natural histor*)

#1 AND #2 AND #3
