# Supplementary material for: Prevalence and natural history of depression after stroke: A systematic review and meta-analysis of observational studies
Source: PLoS Med. 2023 Mar 28;20(3):e1004200. doi: 10.1371/journal.pmed.1004200 (PMC10047522; doi:10.1371/journal.pmed.1004200)
Supplement: S1 Protocol Registry — (DOCX) [file pmed.1004200.s002.docx]

S2 Protocol Registry

At the time of registering the protocol in Prospero, the aim was to produce an updated review and estimate of the prevalence of depression after stroke. During data extraction it became clear there was sufficient literature to complete a more detailed synthesis of the evidence on the natural history of depression in these patients. This was subsequently included as a further aim of this systematic review.
