# Supplementary material for: Prevalence and natural history of depression after stroke: A systematic review and meta-analysis of observational studies
Source: PLoS Med. 2023 Mar 28;20(3):e1004200. doi: 10.1371/journal.pmed.1004200 (PMC10047522; doi:10.1371/journal.pmed.1004200)
Supplement: S1 Table — (DOCX) [file pmed.1004200.s004.docx]

S1Table Prevalence of depression after stroke

| Author&  year | Stroke  type | First-ever stroke | Setting | Country | Assessment criteria | Time  since  stroke | Total  number  assessed | Proportion  with  depression | | |
| --- | --- | --- | --- | --- | --- | --- | --- | --- | --- | --- |
| Kotila et al.1984 | All | N | hospital | Finland | BDI>9 | 3m  12m | 66  63 | 44%  29% | | |
| Wade et al.1987 | All | N | population | UK | WDI>14 | 3w  6m  12m | 379  377  348 | 33%  32%  31% | | |
| Robinson et al.1987 | All-SAH | N | hospital | USA | DSM | 1y  2y | 37  48 | 33%  42% | | |
| Ebrahim et al.1987 | All | N | hospital | UK | GHQ≥12 | 6m | 149 | 23% | | |
| House et al.1991# | All | Y | population | UK | DSM-III/BDI | 1m  6m  12m | 89/76  119/107  112/88 | 25%/32%  22%/32%  15%/16% | | |
| Astrom et al.1993 | All-SAH | N | hospital | Sweden | DSM-III major | Discharge  3m  1y  2y  3y | 76  73  68  57  49 | 25%  31%  16%  19%  29% | | |
| Andersen et al.1994 | All-SAH | N | hospital | Denmark | HDRS>13 | 1m | 209 | 21% | | |
| Burvill et al.1995$ | All | N | population | Australia | DSM-III/PSE | 4m | 294/294 | 23%/20% | | |
| Kotila et al.1998 | All | Y | population | Finland | BDI>9 | 3  12m | 321  311 | 47%  47% | | |
| Herrmann et al. 1998 | All-SAH | N | hospital | Canada | MADRS≥7 | 3  12m | 150  133 | 27%  22% | | |
| Kellermann et al.1999* | All | N | hospital | Hungary | DSM-IV/BS | 1w  after admission | 82/82 | 27%/20% | | |
| Carod-Artal et al.2000 | All-SAH | N | rehabilitation | Spain | HDRS | 1y | 90 | 38% | | |
| Gillen et al.2001 | All | N | rehabilitation | USA | GDS≥15 | 4 d  after rehabilitati-  on | 243 | 13% | | |
| Hayee et al.2001 | All | Y | hospital | Bangladesh | BDI>9 | 3m  12m | 161  156 | 41%  42% | | |
| Aben et al. 2002 | ischemic | Y | hospital | Netherlands | DSM-IV (SCID) | 1m | 190 | 22% | | |
| Tang et al.2002 | All-SAH | Y | rehabilitation | China | DSM-III-R(SCID) | 1m | 157 | 17% | | |
| Appelros et al.2004 | All-SAH | Y | population | Sweden | DSM-IV | 1y | 253 | 27% | | |
| Creed et al.2004 | All | Y | hospital | UK | DSM-IV | 2w  after  admission | 40 | 18% | | |
| Jo ̈nsson et al.2005 | All | Y | population | Sweden | GDS-20≥6 | 16m | 294 | 41% | | |
| Storor et al.2006 | All | N | hospital | Australia | CES-D≥16 | 2w  after admission | 61 | 33% | | |
| Haacke et al.2006 | All | N | hospital | Germany | HADS≥8 | 4y | 63 | 25% | | |
| Paul et al.2006 | All-SAH | Y | population | Australia | IDA≥7 | 5y | 289 | 17% | | |
| Caeiro et al.2006 | All | N | hospital | Portugal | DSM-IV | within4 day | 178 | 46% | | |
| Fure et al.2006 | ischemic | N | hospital | Norway | HADS≥8 | 3-7day | 178 | 14% | | |
| Skånér et al.2007 | All-SAH | Y | population | Sweden | MADRS≥12 | 3m  12m | 92  96 | 55%  59% | | |
| Barker-Collo et al.2007 | All | N | rehabilitation | New Zealand | BDI≥20 | 3m | 73 | 23% | | |
| Brodaty et al.2007 | ischemic | N | hospital | Australia | DSM-IV(SCID) | 3-6m  15m | 158  140 | 12%  21% | | |
| Townend et al.2007 | All | N | hospital | Australia | HADS≥8 | 2-5day  1m  3m | 125  112  105 | 5%  16%  21% | | |
| Saxena et al.2008 | All | N | rehabilitation | Singapore | GDS≥6 | admission  discharge  6m | 200  178  141 | 60%  38%  34% | | |
| Chausson et al.2010 | All | Y | population | France | MADRS≥8 | 5y | 252 | 26% | | |
| Townend et al.2010 | All | N | rehabilitation | UK | DSM-IV | 1m  9m | 89  81 | 33%  30% | | |
| Donnellan et al.2010 | All-SAH | N | hospital | UK | HADS≥8 | 1m  1y | 107  107 | 35%  36% | | |
| Feigin et al.2010 | All | N | population | New Zealand | GHQ-28≥5 | 5y | 418 | 30% | | |
| Sagen et al.2010 | All | N | hospital | Norway | DSM-IV | 4m | 104 | 19% | | |
| Sienkiewicz-Jarosz et al.2010 | All | Y | hospital | Poland | GDS≥6 | 3m | 242 | 34% | | |
| Hackett et al.2010 | All | N | hospital | UK | PSE | 6m | 492 | 26% | | |
| Liman et al.2012 | All | Y | population | Germany | ZRDS>40 | 12m  36m  60m | 705  515  301 | 11%  10%  10% | |  |
| Brown et al.2012 | All | N | hospital | Australia | CES-D≥16 | 2w  3 m  12m | 181  123  146 | 18%  15%  16% | |  |
| Paolucci et al.2012 | ischemic | Y | rehabilitation | Italy | BDI≥10 | admission | 164 | 54% | |  |
| Zhang et al.2012 | All-SAH | N | population | China | DSM-IV | 14d | 1687 | 28% | |  |
| Mutai et al.2013 | All | N | rehabilitation | Japan | GDS≥11 | 1–3 y | 153 | 22% | |  |
| Abdul-sattar et al.2013 | All | N | rehabilitation | Saudi Arabia | GDS-15≥5 | admission | 180 | 63% | |  |
| Ayerbe et al.2013 | All | Y | population | UK | HADS≥8 | 3m  1y  2y  3y  4y  5y  6y  7y  8y  9y  10y  11y  12y  13y  14y  15y | 1101  1233  901  1100  890  658  600  475  392  296  234  183  116  72  46  16 | 33%  29%  30%  31%  30%  30%  30%  32%  29%  36%  35%  30%  32%  39%  30%  31% | |  |
| White et al.2014 | All | N | hospital | Australia | HADS≥8 | within 1w  3m  6m  9m  12m | 134  121  109  109  110 | 22%  29%  22%  28%  20% | |  |
| De Ryck et al.2014 | All | N | hospital | Belgium | CSD≥8 | 1m  3m  6m  12m  18m | 94  170  99  116  156 | 25%  27%  28%  20%  26% | |  |
| Wichowicz et al.2015 | ischemic | Y | hospital | Poland | ICD-10 | 6w | 105 | 23% | |  |
| De Man-Van Ginkel et al.2015 | All-SAH | N | hospital | Netherlands | DSM-IV | 6-8w | 382 | 14% | |  |
| Schöttke et al.2015 | All-SAH | N | rehabilitation | Germany | DSM-IV | 6w | 289 | 31% | |  |
| Ng et al. 2016 | All | N | rehabilitation | Singapore | consensus among the managing team | inpatient | 1277 | 17% |  |  |
| McCarthy et al.2016 | ischemic | N | population | US | CESD-10≥8 | 3m | 322 | 37% |  |  |
| Arba et al. 2016 | ischemic | N | population | Italy,UK,  US,Australia | HADS≥8 | 12m | 2160 | 19% |  |  |
| Yu et al.2016 | ischemic | N | hospital | Australia | HADS≥8 | within 2w  after  discharge | 182 | 16% |  |  |
| Husseini et al.2017 | ischemic | N | population | USA | PHQ-8≥10 | 3m  12m | 1444  1444 | 18%  16% |  |  |
| Mutai et al.2017 | All | N | hospital | Japan | HADS ≥11 | within 2w | 101 | 27% |  |  |
| Barra et al.2017 | All-SAH | N | hospital | Norway | HADS ≥8 | 3m after discharge | 393 | 23% |  |  |
| Stern-Nezer et al.2017 | hemorrhagic | Y | hospital | USA | HDRS>10 | 1y | 89 | 15% |  |  |
| Barker-Collo et al.2017 | ischemic | Y | population | New Zealand | HADS ≥8 | within 2w,  1m  6m  12m | 202  348  346  365 | 13%  12%  10%  10% |  |  |
| Limampai et al.2017 | All | N | rehabilitation | Thailand | HADS≥11 | Discharge  12m | 170  200 | 15%  21% |  |  |
| Broussy et al.2019 | All | N | rehabilitation | France | HADS ≥11 | 1y | 146 | 25% |  |  |
| Bovim et al.2019 | All | N | population | Norway | HADS ≥8 | 3m | 205 | 17% |  |  |
| Kowalska et al.2020 | All | N | population | Poland | PHQ-9 ≥5 | In hospital  3m  12m | 524  388  263 | 55%  59%  55% |  |  |
| Dong et al.2020 | All-SAH | Y | population | USA | PHQ-8 ≥10 | 3m | 786 | 30% |  |  |
| Fournier et al.2020 | ischemic | N | hospital | USA | PHQ-9 ≥5 | In hospital  within 6m | 201  201 | 30%  46% |  |  |
| Roth et al. 2020 | ischemic | Y | population | USA | CES-D≥16 | 9m | 205 | 19% |  |  |
| Ojagbemi et al.2021 | All | Y | hospital | Nigeria | CES-D≥10 | 3m | 150 | 31% |  |  |
| Noushad et al.2021 | All | N | hospital | India | HDRS | In hospital | 81 | 44% |  |  |
| Volz et al. 2021 | ischemic | N | rehabilitation | Germany | DSM-5 | Inpatient  9m  17m  27m  33m | 301  195  179  80  49 | 37%  32%  26%  14%  12% |  |  |
| Williams et al.2021 | All | N | population | UK | HADS≥8 | 6m | 437 | 26% |  |  |
| Ojagbemi et al.2022 | All | Y | population | Ghana and  Nigeria | HRQOLISP | within10d | 1,977 | 26% |  |  |
| Suzuki et al.2022 | All | N | rehabilitation | Japan | NPINH | 1m  3m | 204  204 | 23%  21% |  |  |
| Qawasmeh et al.2022 | All-SAH | N | hospital | Jordan | PHQ-9 ≥5 | 1m after admission  3m | 141  138 | 25%  18% |  |  |
| Stokman-Meiland et al.2022 | All | N | rehabilitation | Netherland | HADS≥8 | 3m  6m  12m | 151  151  151 | 27%  19%  25% |  |  |
| Ytterberg et al.2022 | All | N | hospital | Sweden | HADS≥4 | 6y | 105 | 40% |  |  |
| Shin et al.2022 | All | Y | population | Korean | K-GDS-SF≥8 | 3m | 3215 | 26% |  |  |
| Sagen-Vik et al. 2022 | All | N | hospital | Norway | DSM-5 | 4m  2y | 103  75 | 19%  11% |  |  |
| Ladwig et al. 2022 | ischemic | N | hospital | Germany | DSM-5 | 6m | 183 | 18% |  |  |
| Blo ̈chl et al. 2022 | All | Y | population | UK | CES-D ≥3 | Within 1m  6y | 370  105 | 34%  35% |  |  |

Notes:

No cut off point indicates it was not reported by authors

# Both clinical interview and rating scale are used to assess post-stroke depression. The prevalence of depression at 1m,6m,12m after stroke was 25%,22%,15% respectively by clinical review and was 32%,32%,16% respectively by rating scale.

$ Both clinical interview and rating scale are used to assess post-stroke depression. The prevalence of depression was 23% by clinical review and was 20% by rating scale.

* Both clinical interview and rating scale are used to assess post-stroke depression. The prevalence of depression was 27% by clinical review and was 20% by rating scale.

All-SAH: all stroke subtypes except SAH included

BDI: Beck Depression Inventory

GHQ: General Health Questionnaire
WDI: Wakefield Depression Inventory

DSM: Diagnostic and Statistical Manual Mental Disorders

HDRS: Hamilton Depression Rating Scale

GDS: Geriatric Depression Scale

K-GDS-SF: Korean Geriatric Depression Scale Short Form

CES-D: Centre for Epidemiologic Studies – Depression Scale

MADRS: Montgomery-Åsberg Depression Rating Scale

IDA: Irritability Depression and Anxiety Scale

PSE: Present State Examination

ZRDS: Zung Self Rating Depression Scale

HADS: Hospital Anxiety and Depression Scale

CSD: Cornell Scale for Depression

ICD: International Classification of Diseases

PHQ: Patient Health Questionnaire

BS:13-item Beck Scale

HRQOLISP: Health Related Quality of Life in Stroke patients

NPINH: Neuropsychiatric Inventory-Nursing Home Version

**Reference**

1.Kotila M, Waltimo O, Niemi ML, Laaksonen R, Lempinen M. The profile of recovery from stroke and factors influencing outcome. Stroke. 1984;15(6):1039-44.

2. Wade DT, Legh-Smith J, Hewer RA. Depressed mood after stroke. A community study of its frequency. British Journal of Psychiatry. 1987;151(AUG.):200-5.

3. Robinson RG, Bolduc PL, Price TR. Two-year longitudinal study of poststroke mood disorders: Diagnosis and outcome at one and two years. Stroke. 1987;18(5):837-43.

4. Ebrahim S, Barer D, Nouri F. Affective illness after stroke. British Journal of Psychiatry. 1987;151:52-6.

5. House A, Dennis M, Mogridge L, Warlow C, Hawton K, Jones L. Mood disorders in the year after first stroke. British Journal of Psychiatry. 1991;158(JAN.):83-92.

6. Astrom M, Adolfsson R, Asplund K. Major depression in stroke patients: A 3-year longitudinal study. Stroke. 1993;24(7):976-82.

7. Andersen G, Vestergaard K, Riis JO, Lauritzen L. Incidence of post-stroke depression during the first year in a large unselected stroke population determined using a valid standardized rating scale. Acta Psychiatrica Scandinavica. 1994;90(3):190-5.

8. Burvill PW, Johnson GA, Jamrozik KD, Anderson CS, Stewart-Wynne EG, Chakera TMH. Prevalence of depression after stroke: The Perth Community Stroke Study. British Journal of Psychiatry. 1995;166(MAR.):320-7.

9. Kotila M, Numminen H, Waltimo O, Kaste M. Depression after stroke: Results of the FINNSTROKE study. Stroke. 1998;29(2):368-72.

10. Herrmann N, Black SE, Lawrence J, Szekely C, Szalai JP. The Sunnybrook stroke study - A prospective study of depressive symptoms and functional outcome. Stroke. 1998;29(3):618-24.

11. Kellermann M, Fekete I, Gesztelyi R, Csiba L, Kollar J, Sikula J, et al. Screening for depressive symptoms in the acute phase of stroke. General Hospital Psychiatry. 1999;21(2):116-21.

12. Carod-Artal J, Egido JA, Gonzalez JL, de Seijas EV. Quality of life among stroke survivors evaluated 1 year after stroke - Experience of a stroke unit. Stroke. 2000;31(12):2995-3000.

13. Gillen R, Tennen H, McKee TE, Gernert-Dott P, Affleck G. Depressive symptoms and history of depression predict rehabilitation efficiency in stroke patients. Arch Phys Med Rehabil. 2001;82(12):1645-9.

14. Hayee MA, Akhtar N, Haque A, Rabbani MG. Depression after stroke-analysis of 297 stroke patients. Bangladesh Medical Research Council Bulletin. 2001;27(3):96-102.

15. Aben I, Verhey F, Lousberg R, et al. Validity of the Beck depression inventory, hospital anxiety and depression scale, SCL-90, and Hamilton depression rating scale as screening instruments for depression in stroke patients. Psychosomatics 2002;43:386–93.

16. Tang WK, Ungvari GS, Chiu HFK, Sze KH, Woo J, Kay R. Psychiatric morbidity in first time stroke patients in Hong Kong: A pilot study in a rehabilitation unit. Australian and New Zealand Journal of Psychiatry. 2002;36(4):544-9.

17. Appelros P, Viitanen M. Prevalence and predictors of depression at one year in a Swedish population-based cohort with first-ever stroke. Journal of Stroke and Cerebrovascular Diseases. 2004;13(2):52-7.

18. Creed A, Swanwick G, O'Neill D. Screening for post stroke depression in patients with acute stroke including those with communication disorders. International Journal of Geriatric Psychiatry. 2004;19(6):595-7.

19. Jonsson AC, Lindgren I, Hallstrom B, Norrving B, Lindgren A. Determinants of quality of life in stroke survivors and their informal caregivers. Stroke. 2005;36(4):803-8.

20. Storor DL, Byrne GJ. Pre-morbid personality and depression following stroke. International Psychogeriatrics. 2006;18(3):457-69.

21.Haacke C, Althaus A, Spottke A, Siebert U, Back T, Dodel R. Long-term outcome after stroke - Evaluating health-related quality of life using utility measurements. Stroke. 2006;37(1):193-8.

22. Paul SL, Dewey HM, Sturm JW, Macdonell RAL, Thrift AG. Prevalence of depression and use of antidepressant medication at 5-years poststroke in the North East Melbourne stroke incidence study. Stroke. 2006;37(11):2854-5.

23. Caeiro L, Ferro JM, Santos CO, Figueira ML. Depression in acute stroke. Journal of Psychiatry and Neuroscience. 2006;31(6):377-83.

24. Fure B, Wyller TB, Engedal K, Thommessen B. Emotional symptoms in acute ischemic stroke. International Journal of Geriatric Psychiatry. 2006;21(4):382-7.

25. Skaner Y, Nilsson GH, Sundquist K, Hassler E, Krakau I. Self-rated health, symptoms of depression and general symptoms at 3 and 12 months after a first-ever stroke: A municipality-based study in Sweden. BMC Family Practice. 2007;8.

26. Barker-Collo S, Krishnamurthi R, Witt E, Theadom A, Starkey N, Barber PA, et al. Depression and Anxiety Across the First Year After Ischemic Stroke: Findings from a Population-Based New Zealand ARCOS-IV Study. Brain Impair. 2017;18(3):265-76

27. Brodaty H, Withall A, Altendorf A, Sachdev PS. Rates of depression at 3 and 15 months poststroke and their relationship with cognitive decline: The Sydney stroke study. American Journal of Geriatric Psychiatry. 2007;15(6):477-86.

28. Townend BS, Whyte S, Desborough T, Crimmins D, Markus R, Levi C, et al. Longitudinal prevalence and determinants of early mood disorder post-stroke. J Clin Neurosci. 2007;14(5):429-34.

29. Saxena SK, Ng TP, Yong D, Fong NP, Koh G. Subthreshold depression and cognitive impairment but not demented in stroke patients during their rehabilitation. Acta Neurologica Scandinavica. 2008;117(2):133-40.

30. Chausson N, Olindo S, Cabre P, Saint-Vil M, Smadja D. Five-year outcome of a stroke cohort in martinique, French West Indies: Etude realisee en martinique et centree sur l'incidence des accidents vasculaires cerebraux, part 2. Stroke. 2010;41(4):594-9.

31. Townend E, Tinson D, Kwan J, Sharpe M. 'Feeling sad and useless': an investigation into personal acceptance of disability and its association with depression following stroke. Clin Rehabil. 2010;24(6):555-64.

32. Donnellan C, Hickey A, Hevey D, O'Neill D. Effect of mood symptoms on recovery one year after stroke. International Journal of Geriatric Psychiatry. 2010;25(12):1288-95.

33. Feigin VL, Barker-Collo S, Parag V, Senior H, Lawes CMM, Ratnasabapathy Y, et al. Auckland Stroke Outcomes Study: Part 1: Gender, stroke types, ethnicity, and functional outcomes 5 years poststroke. Neurology. 2010;75(18):1597-607.

34. Sagen U, Finset A, Moum T, Morland T, Vik TG, Nagy T, et al. Early detection of patients at risk for anxiety, depression and apathy after stroke. General Hospital Psychiatry. 2010;32(1):80-5.

35. Sienkiewicz-Jarosz H, Milewska D, Bochynska A, Chelmniak A, Dworek N, Kasprzyk K, et al. Predictors of depressive symptoms in patients with stroke - a three-month follow-up. Neurol Neurochir Pol. 2010;44(1):13-20.

36. Hackett ML, Hill KM, Hewison J, Anderson CS, House AO. Stroke Survivors Who Score below Threshold on Standard Depression Measures May Still Have Negative Cognitions of Concern. Stroke. 2010;41(3):478-81.

37. Liman TG, Heuschmann PU, Endres M, Floel A, Schwab S, Kolominsky-Rabas PL. Impact of low mini-mental status on health outcome up to 5 years after stroke: the Erlangen Stroke Project. J Neurol. 2012;259(6):1125-30.

38. Brown C, Hasson H, Thyselius V, Almborg AH. Post-stroke depression and functional independence: A conundrum. Acta Neurologica Scandinavica. 2012;126(1):45-51.

39. Paolucci S, Di Vita A, Massicci R, Traballesi M, Bureca I, Matano A, et al. Impact of participation on rehabilitation Results: A multivariate study. European Journal of Physical and Rehabilitation Medicine. 2012;48(3):455-66.

40. Zhang N, Wang CX, Wang AX, Bai Y, Zhou Y, Wang YL, et al. Time course of depression and one-year prognosis of patients with stroke in mainland China. CNS Neuroscience & Therapeutics. 2012;18(6):475-81.

41. Mutai H, Furukawa T, Araki K, Misawa K, Hanihara T. Long-term outcome in stroke survivors after discharge from a convalescent rehabilitation ward. Psychiatry and Clinical Neurosciences. 2013;67(6):434-40.

42. Abdul-sattar AB, Godab T. Predictors of functional outcome in Saudi Arabian patients with stroke after inpatient rehabilitation. Neurorehabilitation. 2013;33(2):209-16.

43. Ayerbe L, Ayis S, Crichton S, Wolfe CDA, Rudd AG. The natural history of depression up to 15 years after stroke: The South London stroke register. Stroke. 2013;44(4):1105-10.

44. White JH, Attia J, Sturm J, Carter G, Magin P. Predictors of depression and anxiety in community dwelling stroke survivors: a cohort study. Disability and rehabilitation. 2014;36(23):1975-82.

45. De Ryck A, Fransen E, Brouns R, Geurden M, Peij D, Marien P, et al. Poststroke depression and its multifactorial nature: Results from a prospective longitudinal study. Journal of the Neurological Sciences. 2014;347(1-2):159-66.

46. Wichowicz HM, Gasecki D, Lass P, Landowski J, Swierkocka M, Wisniewski G, et al. Clinical utility of chosen factors in predicting post-stroke depression: a one year follow-up. Psychiatr Pol. 2015;49(4):683-96.

47. De Man-Van Ginkel JM, Hafsteinsdottir TB, Lindeman E, Geerlings MI, Grobbee DE, Schuurmans MJ. Clinical Manifestation of Depression after Stroke: Is It Different from Depression in Other Patient Populations? PLoS ONE. 2015;10(12).

48. Schottke H, Giabbiconi CM. Post-stroke depression and post-stroke anxiety: Prevalence and predictors. International Psychogeriatrics. 2015;27(11):1805-12.

49. Ng YS, Tan KH, Chen C, Senolos GC, Koh GC. How Do Recurrent and First-Ever Strokes Differ in Rehabilitation Outcomes? American journal of physical medicine & rehabilitation. 2016;95(10):709-17.

50. McCarthy MJ, Sucharew HJ, Alwell K, Moomaw CJ, Woo D, Flaherty ML, et al. Age, subjective stress, and depression after ischemic stroke. Journal of behavioral medicine. 2016;39(1):55-64.

51. Arba F, Ali M, Quinn TJ, Hankey GJ, Lees KR, Inzitari D. Lacunar Infarcts, Depression, and Anxiety Symptoms One Year after Stroke. Journal of Stroke and Cerebrovascular Diseases. 2016;25(4):831-4.

52. Yu S, Arima H, Bertmar C, Hirakawa Y, Priglinger M, Evans K, et al. Depression but not anxiety predicts recurrent cerebrovascular events. Acta Neurologica Scandinavica. 2016;134(1):29-34.

53. El Husseini N, Goldstein LB, Peterson ED, Zhao X, Olson DM, Williams JW, Jr., et al. Depression Status Is Associated with Functional Decline Over 1-Year Following Acute Stroke. Journal of Stroke & Cerebrovascular Diseases. 2017;26(7):1393-9.

54. Mutai H, Furukawa T, Houri A, Suzuki A, Hanihara T. Factors associated with multidimensional aspect of post-stroke fatigue in acute stroke period. Asian Journal of Psychiatry. 2017;26:1-5.

55. Barra M, Evensen GSH, Valeberg BT. Cues and clues predicting presence of symptoms of depression in stroke survivors. J Clin Nurs. 2017;26(3-4):546-56.

56. Stern-Nezer S, Eyngorn I, Mlynash M, Snider RW, Venkatsubramanian C, Wijman CAC, et al. Depression one year after hemorrhagic stroke is associated with late worsening of outcomes. Neurorehabilitation. 2017;41(1):179-87.

57. Barker-Collo S, Krishnamurthi R, Witt E, Theadom A, Starkey N, Barber PA, et al. Depression and Anxiety Across the First Year After Ischemic Stroke: Findings from a Population-Based New Zealand ARCOS-IV Study. Brain Impair. 2017;18(3):265-76

58. Limampai P, Wongsrithep W, Kuptniratsaikul V. Depression after stroke at 12-month follow-up: a multicenter study. International Journal of Neuroscience. 2017;127(10):887-92.

59. Broussy S, Saillour-Glenisson F, Garcia-Lorenzo B, Rouanet F, Lesaine E, Maugeais M, et al. Sequelae and quality of life in patients living at home 1 year after a stroke managed in stroke units. Frontiers in Neurology. 2019;10(AUG).

60. Bovim MR, Lndredavik B, Hokstad A, Cumming T, Bernhardt J, Askim T. Relationship between pre-stroke physical activity and symptoms of post-stroke anxiety and depression. J Rehabil Med. 2019;51(10):755-60.

61. Kowalska K, Dros J, Mazurek M, Pasinska P, Gorzkowska A, Klimkowicz-Mrowiec A. Delirium post-stroke: Short-and long-term effect on depression, anxiety, apathy and aggression (research study-part of propolis study). Journal of Clinical Medicine. 2020;9(7):1-11

62. Dong L, Sanchez BN, Skolarus LE, Stulberg E, Morgenstern LB, Lisabeth LD. Sex difference in prevalence of depression after stroke. Neurology. 2020;94(19):e1973-e83.

63. Fournier LE, Beauchamp JES, Zhang X, Bonojo E, Love M, Cooksey G, et al. Assessment of the Progression of Poststroke Depression in Ischemic Stroke Patients Using the Patient Health Questionnaire-9. Journal of Stroke & Cerebrovascular Diseases. 2020;29(4):8.

64. Roth DL, Haley WE, Sheehan OC, Liu C, Clay OJ, Rhodes JD, et al. Depressive Symptoms After Ischemic Stroke Population-Based Comparisons of Patients and Caregivers With Matched Controls. Stroke. 2020;51(1):54-60.

65. Ojagbemi A, Bello T, Owolabi M, Baiyewu O. Prevalence, predictors, and prognoses of prestroke neuropsychiatric symptoms at 3 months poststroke. International Psychogeriatrics. 2021;33(8):827-34.

66. Noushad N, Sachita S, Varughese SA, Joy SK, Jose S. Post stroke depression and anxiety: Prevalance and correlates. Asian Journal of Pharmaceutical and Clinical Research. 2021;14(9):142-7.

67. Volz M, Ladwig S, Werheid K. Gender differences in post-stroke depression: A longitudinal analysis of prevalence, persistence and predictive value of known risk factors. Neuropsychological rehabilitation. 2021;31(1):1-17.

68. Williams OA, Demeyere N. Association of Depression and Anxiety With Cognitive Impairment 6 Months After Stroke. Neurology. 2021;96(15):E1966-E74.

69. Ojagbemi A, Akinyemi J, Wahab K, Owolabi L, Arulogun O, Akpalu J, et al. Pre-Stroke Depression in Ghana and Nigeria: Prevalence, Predictors and Association With Poststroke Depression. Journal of Geriatric Psychiatry and Neurology. 2022;35(1):121-7.

70. Suzuki, A., et al. The Prevalence and Course of Neuropsychiatric Symptoms in Stroke Patients Impact Functional Recovery During in-Hospital Rehabilitation.Topics in Stroke Rehabilitation 29(1) (2022): 1-8.

71. Al Qawasmeh M, Aldabbour B, Abuabada A, Abdelrahman K, Elamassie S, Khweileh M, et al. Prevalence, Severity, and Predictors of Poststroke Depression in a Prospective Cohort of Jordanian Patients. Stroke Research and Treatment. 2022;2022:6506326.

72. Stokman-Meiland DCM, Groeneveld IF, Arwert HJ, van der Pas SL, Meesters JJL, Mishre RRD, et al. The course of depressive symptoms in the first 12 months post-stroke and its association with unmet needs. Disability and Rehabilitation. 2022;44(3):428-35.

73. Ytterberg C, Cegrell L, von Koch L, Wiklander M. Depression symptoms 6 years after stroke are associated with higher perceived impact of stroke, limitations in ADL and restricted participation. Scientific Reports. 2022;12(1).

74. Shin M, Sohn MK, Lee J, Kim DY, Shin YI, Oh GJ, et al. Post-Stroke Depression and Cognitive Aging: A Multicenter, Prospective Cohort Study. Journal of Personalized Medicine. 2022;12(3).

75. Sagen-Vik U, Finset A, Moum T, Vik TG, Dammen T. The longitudinal course of anxiety, depression and apathy through two years after stroke. Journal of Psychosomatic Research. 2022;162.

76. Ladwig S, Ziegler M, Sudmeyer M, Werheid K. The Post-Stroke Depression Risk Scale (PoStDeRiS): Development of an Acute-Phase Prediction Model for Depression 6 Months After Stroke. Journal of the Academy of Consultation-Liaison Psychiatry. 2022;63(2):144-52.

77. Blochl M, Nestler S. Long-term Changes in Depressive Symptoms before and after Stroke. Neurology. 2022;99(7):E720-E9.
