# Supplementary material for: Prevalence and natural history of depression after stroke: A systematic review and meta-analysis of observational studies
Source: PLoS Med. 2023 Mar 28;20(3):e1004200. doi: 10.1371/journal.pmed.1004200 (PMC10047522; doi:10.1371/journal.pmed.1004200)
Supplement: S2 Table — (DOCX) [file pmed.1004200.s005.docx]

S2 Table Natural history of post-stroke depression

| Author & year | Time  Since  stroke | Number  of  assessed  patients | Number  Of depressed  at each  assessment | Proportion  of  recovery  among  depressed  at initial  assessment | Proportion  of  persistent  cases  among  depressed  at initial  assessment | Proportion  of  incident cases | cumulative incidence  during  follow-up | proportion of  incident case  within  3 months  among  depressed  within 1y |
| --- | --- | --- | --- | --- | --- | --- | --- | --- |
| Stokman-Meiland et al. 2022* | 3m  6m  1y | 151  151  151 | 41  29  37 | 37% | 44% | 5% | 37% | 73% |
| Suzuki et al.2022* | 1m  3-5m | 204  204 | 46  42 | 39% | 61% | 7% | 29% | 77% |
| Noushad  et al.2021  ^a^ | Hospital  3m | 81 | 36 | 26% | 74% | / | / | / |
| Fournier *  et al.2020 | Hospital  6m | 201  201 | 60  92 | 30% | 70% | 25% | 55% | 55% |
| Limampai  et al.  2017* | 1m  1y | 170 | 25 | 68% | 32% | 16% | 31% | 47% |
| Husseini et al.2017  * | 3m  1y | 1444  1444 | 260  238 | 48% | 52% | 7% | 25% | 72% |
| Barker-Collo et al.2017^b^ | 2w  1m  6m  1y | 208  353  346  365 | 26  43  33  35 | 58% | 31% | / | / | / |
| Jorgensen et al.2016^d^ | 0-3m  3-12m  1-2y | 135417  99752  82911 |  | / | / | 13%  11%  6% | 21%  25% | 61% |
| Wichowicz et al.  2015^d^ | 6w  3m  6m  1y | 105 | 24  15  11  4 | / | / | 1% | 28% | 83% |
| Ayerbe et al.2013* | 3m  1y  2y  3y  4y  5y  6y  7y  8y  9y  10y  11y  12y  13y  14y  15y | 750  750  450  329  249  154  87  44  36  27  14  11  5  N/A  N/A  N/A | 232  198 | 50% | 50% | 11%  9%  5%  6%  2%  0  8%  0  4%  0  0  0  0  0  0 | 42% | 74% |
| Zhang et al.2012^c^ | 14d  3m  6m  1y | 1687  1208  1073  1034 | 479  135  39  52 | / | / | 28%  11%  4%  5% | 42% | 87% |
| Townend et al.2010^d^ | 1m  9m | 89  81 | 29  24 | / | 66% | 6% | 38% | 85% |
| Donnellan et al.2010  * | 1m  1y | 107  107 | 37  39 | 35% | 65% | 14% | 49% | 71% |
| Townend et al.2007 | 2-5d  1m  3m | 125  112  105 | 6  18  22 | 50% | 50% | / | / | / |
| Brodaty et al.2007* | 3m  15m | 135  135 | 19  29 | 37% | 58% | 13% | 27% | 51% |
| Aben et al. 2002^c^ | 1m  3m  6m  9m  1y | 190  137.5  117  107  98 | 41  7  7  6  7 | / | / | 21.6%  5.1%  6.0%  5.6%  7.1% | 38.7% | 71% |
| Tang et al.2002^a^ | 1m  4-6m | 157 | 27 | 26% | 67% | / | / | / |
| Herrmann et al. 1998  ^b^ | 3m  1y | 150  136 | 40 | 30% | 45% | / | / | / |
| Burvill et al.1995^a^ | 4m  1y | 248  234 | 69 | 52% | 41% | / | / | / |
| Andersen et al.1994  ^c^ | 1m  3m  6m  12m | 209  204  199  191 | 43  24  12  6 | / | / | 21%  15%  9%  5% | 41% | 79% |
| Astrom et al.1993^a^ | Hospital  3m  1year  2year  3year | 76  73  68  57  49 | 19  23  11  11  14 | 56% | 37% | / | / | / |
| House et al.1991* | 1m  6m  1y | 60  60  60 | 14  16 | 8(57%) | 6(43%) | 10(17%) | 24(40%) | 58% |
| Wade et al.1987* | 3w  6m  1y | 255  255  255 | 80 | / | 55% | 10% | 48% | 60% |
| Robinson et al.  1987* | 1m  1y | 37  37 | 12  12 | 5(42%) | 7(58%) | 5(14%) | 17(46%) | 71% |

Notes:

N/A. no observations at corresponding assessment time

/. Information is not given

* studies with analysis in participants completed follow-up at all prespecified time points

1. Proportion of persistence or recovery was calculated as the percentage of patients with/without depression among initially depressed patients included in the follow-up assessment.
2. Proportion of persistence/ recovery was calculated as the percentages with/without depression at the follow up time point from the total number of depressed patients at initial assessment
3. Proportion of incident cases was calculated as the percentage of patients not rated as depressed
4. Proportion of incident cases was calculated as the percentage of stroke survivors at corresponding assessment.

**Reference**

1.Stokman-Meiland DCM, Groeneveld IF, Arwert HJ, van der Pas SL, Meesters JJL, Mishre RRD, et al. The course of depressive symptoms in the first 12 months post-stroke and its association with unmet needs. Disability and Rehabilitation. 2022;44(3):428-35.

2. Suzuki, A., et al. The Prevalence and Course of Neuropsychiatric Symptoms in Stroke Patients Impact Functional Recovery During in-Hospital Rehabilitation.Topics in Stroke Rehabilitation 29(1) (2022): 1-8.

3. Noushad N, Sachita S, Varughese SA, Joy SK, Jose S. Post stroke depression and anxiety: Prevalance and correlates. Asian Journal of Pharmaceutical and Clinical Research. 2021;14(9):142-7.

4. Fournier LE, Beauchamp JES, Zhang X, Bonojo E, Love M, Cooksey G, et al. Assessment of the Progression of Poststroke Depression in Ischemic Stroke Patients Using the Patient Health Questionnaire-9. Journal of Stroke & Cerebrovascular Diseases. 2020;29(4):8.

5. Limampai P, Wongsrithep W, Kuptniratsaikul V. Depression after stroke at 12-month follow-up: a multicenter study. International Journal of Neuroscience. 2017;127(10):887-92.

6. El Husseini N, Goldstein LB, Peterson ED, Zhao X, Olson DM, Williams JW, Jr., et al. Depression Status Is Associated with Functional Decline Over 1-Year Following Acute Stroke. Journal of Stroke & Cerebrovascular Diseases. 2017;26(7):1393-9.

7. Barker-Collo SL. Depression and anxiety 3 months post stroke: Prevalence and correlates. Archives of Clinical Neuropsychology. 2007;22(4):519-31.

8. Jorgensen TS, Wium-Andersen IK, Wium-Andersen MK, Jorgensen MB, Prescott E, Maartensson S, et al. Incidence of Depression After Stroke, and Associated Risk Factors and Mortality Outcomes, in a Large Cohort of Danish Patients. JAMA Psychiatry. 2016;73(10):1032-40.

9. Wichowicz HM, Gasecki D, Lass P, Landowski J, Swierkocka M, Wisniewski G, et al. Clinical utility of chosen factors in predicting post-stroke depression: a one year follow-up. Psychiatr Pol. 2015;49(4):683-96.

10. Ayerbe L, Ayis S, Crichton S, Wolfe CDA, Rudd AG. The natural history of depression up to 15 years after stroke: The South London stroke register. Stroke. 2013;44(4):1105-10.

11. Zhang N, Wang CX, Wang AX, Bai Y, Zhou Y, Wang YL, et al. Time course of depression and one-year prognosis of patients with stroke in mainland China. CNS Neuroscience & Therapeutics. 2012;18(6):475-81.

12. Townend E, Tinson D, Kwan J, Sharpe M. 'Feeling sad and useless': an investigation into personal acceptance of disability and its association with depression following stroke. Clin Rehabil. 2010;24(6):555-64.

13. Donnellan C, Hickey A, Hevey D, O'Neill D. Effect of mood symptoms on recovery one year after stroke. International Journal of Geriatric Psychiatry. 2010;25(12):1288-95.

14. Townend BS, Whyte S, Desborough T, Crimmins D, Markus R, Levi C, et al. Longitudinal prevalence and determinants of early mood disorder post-stroke. J Clin Neurosci. 2007;14(5):429-34.

15. Brodaty H, Withall A, Altendorf A, Sachdev PS. Rates of depression at 3 and 15 months poststroke and their relationship with cognitive decline: The Sydney stroke study. American Journal of Geriatric Psychiatry. 2007;15(6):477-86.

16. Aben I, Verhey F, Lousberg R, et al. Validity of the Beck depression inventory, hospital anxiety and depression scale, SCL-90, and Hamilton depression rating scale as screening instruments for depression in stroke patients. Psychosomatics 2002;43:386–93.

17. Tang WK, Ungvari GS, Chiu HFK, Sze KH, Woo J, Kay R. Psychiatric morbidity in first time stroke patients in Hong Kong: A pilot study in a rehabilitation unit. Australian and New Zealand Journal of Psychiatry. 2002;36(4):544-9.

18. Herrmann N, Black SE, Lawrence J, Szekely C, Szalai JP. The Sunnybrook stroke study - A prospective study of depressive symptoms and functional outcome. Stroke. 1998;29(3):618-19. Burvill PW, Johnson GA, Jamrozik KD, Anderson CS, Stewart-Wynne EG, Chakera TMH. Prevalence of depression after stroke: The Perth Community Stroke Study. British Journal of Psychiatry. 1995;166(MAR.):320-7.

20. Andersen G, Vestergaard K, Riis JO, Lauritzen L. Incidence of post-stroke depression during the first year in a large unselected stroke population determined using a valid standardized rating scale. Acta Psychiatrica Scandinavica. 1994;90(3):190-5.

21. Astrom M, Adolfsson R, Asplund K. Major depression in stroke patients: A 3-year longitudinal study. Stroke. 1993;24(7):976-82.

22. House A, Dennis M, Mogridge L, Warlow C, Hawton K, Jones L. Mood disorders in the year after first stroke. British Journal of Psychiatry. 1991;158(JAN.):83-92.

23. Wade DT, Legh-Smith J, Hewer RA. Depressed mood after stroke. A community study of its frequency. British Journal of Psychiatry. 1987;151(AUG.):200-5.

24. Robinson RG, Bolduc PL, Price TR. Two-year longitudinal study of poststroke mood disorders: Diagnosis and outcome at one and two years. Stroke. 1987;18(5):837-43.
