# Supplementary material for: Prevalence and natural history of depression after stroke: A systematic review and meta-analysis of observational studies
Source: PLoS Med. 2023 Mar 28;20(3):e1004200. doi: 10.1371/journal.pmed.1004200 (PMC10047522; doi:10.1371/journal.pmed.1004200)
Supplement: S2 Fig — (PDF) [file pmed.1004200.s008.pdf]

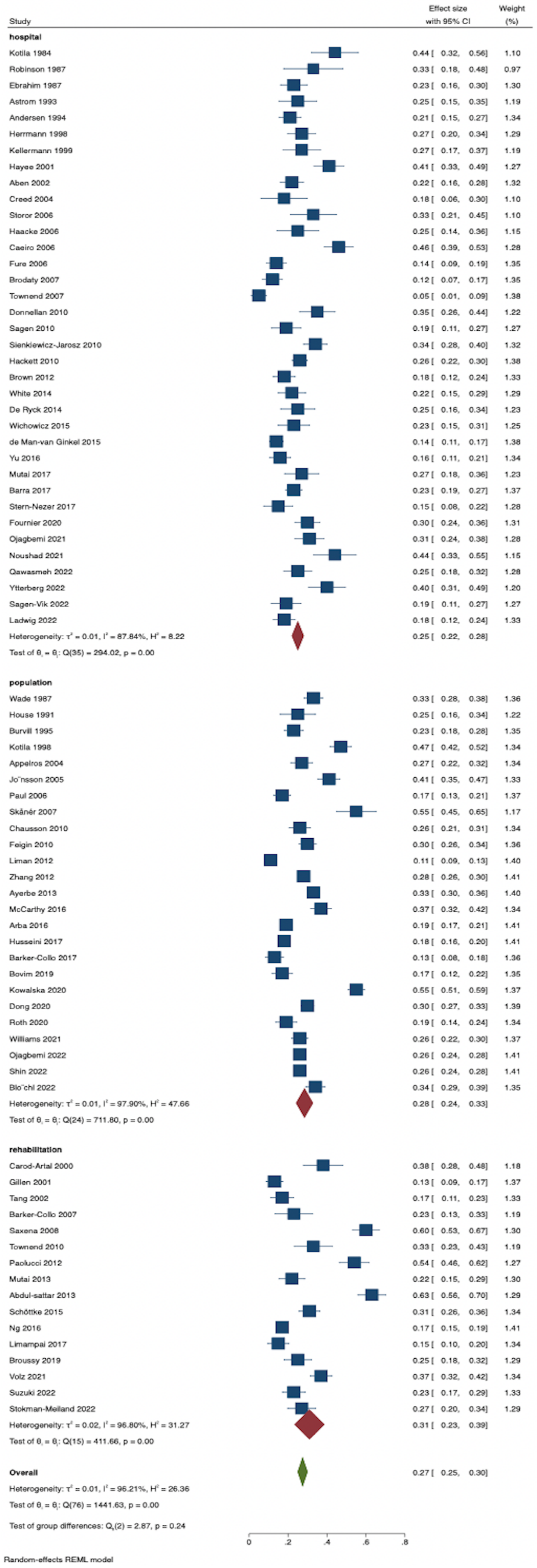

Study
Effect size with 95% CI
Weight (%)
hospital
Kotila 1984 0.44 [0.32, 0.56] 1.10
Robinson 1987 0.33 [0.18, 0.48] 0.97
Ebrahim 1987 0.23 [0.16, 0.30] 1.30
Astrom 1993 0.25 [0.15, 0.35] 1.19
Andersen 1994 0.21 [0.15, 0.27] 1.34
Herrmann 1998 0.27 [0.20, 0.34] 1.29
Kellermann 1999 0.27 [0.17, 0.37] 1.19
Hayee 2001 0.41 [0.33, 0.49] 1.27
Aben 2002 0.22 [0.16, 0.28] 1.32
Creed 2004 0.18 [0.06, 0.30] 1.10
Storor 2006 0.33 [0.21, 0.45] 1.10
Haacke 2006 0.25 [0.14, 0.36] 1.15
Caeiro 2006 0.46 [0.39, 0.53] 1.28
Fure 2006 0.14 [0.09, 0.19] 1.35
Brodaty 2007 0.12 [0.07, 0.17] 1.35
Townend 2007 0.05 [0.01, 0.09] 1.38
Donnellan 2010 0.35 [0.26, 0.44] 1.22
Sagen 2010 0.19 [0.11, 0.27] 1.27
Sienkiewicz-Jarosz 2010 0.34 [0.28, 0.40] 1.32
Hackett 2010 0.26 [0.22, 0.30] 1.38
Brown 2012 0.18 [0.12, 0.24] 1.33
White 2014 0.22 [0.15, 0.29] 1.29
De Ryck 2014 0.25 [0.16, 0.34] 1.23
Wichowicz 2015 0.23 [0.15, 0.31] 1.25
de Man-van Ginkel 2015 0.14 [0.11, 0.17] 1.38
Yu 2016 0.16 [0.11, 0.21] 1.34
Mutai 2017 0.27 [0.18, 0.36] 1.23
Barra 2017 0.23 [0.19, 0.27] 1.37
Stern-Nezer 2017 0.15 [0.08, 0.22] 1.28
Fournier 2020 0.30 [0.24, 0.36] 1.31
Ojagbemi 2021 0.31 [0.24, 0.38] 1.28
Noushad 2021 0.44 [0.33, 0.55] 1.15
Qawasmeh 2022 0.25 [0.18, 0.32] 1.28
Ytterberg 2022 0.40 [0.31, 0.49] 1.20
Sagen-Vik 2022 0.19 [0.11, 0.27] 1.27
Ladwig 2022 0.18 [0.12, 0.24] 1.33
Heterogeneity: τ^2 = 0.01, I^2 = 87.84%, H^2 = 8.22 0.25 [0.22, 0.28]
Test of $\theta_i = \theta_j$: Q(35) = 294.02, p = 0.00
population
Wade 1987 0.33 [0.28, 0.38] 1.36
House 1991 0.25 [0.16, 0.34] 1.22
Burvill 1995 0.23 [0.18, 0.28] 1.35
Kotila 1998 0.47 [0.42, 0.52] 1.34
Appelros 2004 0.27 [0.22, 0.32] 1.34
Jo¨nsson 2005 0.41 [0.35, 0.47] 1.33
Paul 2006 0.17 [0.13, 0.21] 1.37
Skånér 2007 0.55 [0.45, 0.65] 1.17
Chausson 2010 0.26 [0.21, 0.31] 1.34
Feigin 2010 0.30 [0.26, 0.34] 1.36
Liman 2012 0.11 [0.09, 0.13] 1.40
Zhang 2012 0.28 [0.26, 0.30] 1.41
Ayerbe 2013 0.33 [0.30, 0.36] 1.40
McCarthy 2016 0.37 [0.32, 0.42] 1.34
Arba 2016 0.19 [0.17, 0.21] 1.41
Husseini 2017 0.18 [0.16, 0.20] 1.41
Barker-Collo 2017 0.13 [0.08, 0.18] 1.36
Bovim 2019 0.17 [0.12, 0.22] 1.35
Kowalska 2020 0.55 [0.51, 0.59] 1.37
Dong 2020 0.30 [0.27, 0.33] 1.39
Roth 2020 0.19 [0.14, 0.24] 1.34
Williams 2021 0.26 [0.22, 0.30] 1.37
Ojagbemi 2022 0.26 [0.24, 0.28] 1.41
Shin 2022 0.26 [0.24, 0.28] 1.41
Blo¨chl 2022 0.34 [0.29, 0.39] 1.35
Heterogeneity: τ^2 = 0.01, I^2 = 97.90%, H^2 = 47.66 0.28 [0.24, 0.33]
Test of $\theta_i = \theta_j$: Q(24) = 711.80, p = 0.00
rehabilitation
Carod-Artal 2000 0.38 [0.28, 0.48] 1.18
Gillen 2001 0.13 [0.09, 0.17] 1.37
Tang 2002 0.17 [0.11, 0.23] 1.33
Barker-Collo 2007 0.23 [0.13, 0.33] 1.19
Saxena 2008 0.60 [0.53, 0.67] 1.30
Townend 2010 0.33 [0.23, 0.43] 1.19
Paolucci 2012 0.54 [0.46, 0.62] 1.27
Mutai 2013 0.22 [0.15, 0.29] 1.30
Abdul-sattar 2013 0.63 [0.56, 0.70] 1.29
Schöttke 2015 0.31 [0.26, 0.36] 1.34
Ng 2016 0.17 [0.15, 0.19] 1.41
Limampai 2017 0.15 [0.10, 0.20] 1.34
Broussy 2019 0.25 [0.18, 0.32] 1.29
Volz 2021 0.37 [0.32, 0.42] 1.34
Suzuki 2022 0.23 [0.17, 0.29] 1.33
Stokman-Meiland 2022 0.27 [0.20, 0.34] 1.29
Heterogeneity: τ^2 = 0.02, I^2 = 96.80%, H^2 = 31.27 0.31 [0.23, 0.39]
Test of $\theta_i = \theta_j$: Q(15) = 411.66, p = 0.00
Overall 0.27 [0.25, 0.30]
Heterogeneity: τ^2 = 0.01, I^2 = 96.21%, H^2 = 26.36
Test of $\theta_i = \theta_j$: Q(76) = 1441.63, p = 0.00
Test of group differences: $Q_b(2)$ = 2.87, p = 0.24
0
.2
.4
.6
.8
Random-effects REML model

Reference

1.Kotila M, Waltimo O, Niemi ML, Laaksonen R, Lempinen M. The profile of recovery from stroke and factors influencing outcome. Stroke. 1984;15(6):1039-44.

2. Robinson RG, Bolduc PL, Price TR. Two-year longitudinal study of poststroke mood disorders: Diagnosis and outcome at one and two years. Stroke. 1987;18(5):837-43.

3.Ebrahim S, Barer D, Nouri F. Affective illness after stroke. British Journal of Psychiatry. 1987;151:52-6.

4. Astrom M, Adolfsson R, Asplund K. Major depression in stroke patients: A 3-year longitudinal study. Stroke. 1993;24(7):976-82.

5.Andersen G, Vestergaard K, Riis JO, Lauritzen L. Incidence of post-stroke depression during the first year in a large unselected stroke population determined using a valid standardized rating scale. Acta Psychiatrica Scandinavica. 1994;90(3):190-5.

6.Herrmann N, Black SE, Lawrence J, Szekely C, Szalai JP. The Sunnybrook stroke study - A prospective study of depressive symptoms and functional outcome. Stroke. 1998;29(3):618-24.

7.Kellermann M, Fekete I, Gesztelyi R, Csiba L, Kollar J, Sikula J, et al. Screening for depressive symptoms in the acute phase of stroke. General Hospital Psychiatry. 1999;21(2):116-21.

8.Hayee MA, Akhtar N, Haque A, Rabbani MG. Depression after stroke-analysis of 297 stroke patients. Bangladesh Medical Research Council Bulletin. 2001;27(3):96-102.

9.Aben I, Verhey F, Lousberg R, et al. Validity of the Beck depression inventory, hospital anxiety and depression scale, SCL-90, and Hamilton depression rating scale as screening instruments for depression in stroke patients. Psychosomatics 2002;43:386–93.

10.Creed A, Swanwick G, O'Neill D. Screening for post stroke depression in patients with acute stroke including those with communication disorders. International Journal of Geriatric Psychiatry. 2004;19(6):595-7.

11. Storor DL, Byrne GJ. Pre-morbid personality and depression following stroke. International Psychogeriatrics. 2006;18(3):457-69.

12. Haacke C, Althaus A, Spottke A, Siebert U, Back T, Dodel R. Long-term outcome after stroke - Evaluating health-related quality of life using utility measurements. Stroke. 2006;37(1):193-8.

13. Caeiro L, Ferro JM, Santos CO, Figueira ML. Depression in acute stroke. Journal of Psychiatry and Neuroscience. 2006;31(6):377-83.
14. Fure B, Wyller TB, Engedal K, Thommessen B. Emotional symptoms in acute ischemic stroke. International Journal of Geriatric Psychiatry. 2006;21(4):382-7.
15.Brodaty H, Withall A, Altendorf A, Sachdev PS. Rates of depression at 3 and 15 months poststroke and their relationship with cognitive decline: The Sydney stroke study. American Journal of Geriatric Psychiatry. 2007;15(6):477-86.
16.Townend BS, Whyte S, Desborough T, Crimmins D, Markus R, Levi C, et al. Longitudinal prevalence and determinants of early mood disorder post-stroke. J Clin Neurosci.
17. Donnellan C, Hickey A, Hevey D, O'Neill D. Effect of mood symptoms on recovery one year after stroke. International Journal of Geriatric Psychiatry. 2010;25(12):1288-95.
18. Sagen U, Finset A, Moum T, Morland T, Vik TG, Nagy T, et al. Early detection of patients at risk for anxiety, depression and apathy after stroke. General Hospital Psychiatry. 2010;32(1):80-5.
19. Sienkiewicz-Jarosz H, Milewska D, Bochynska A, Chelmniak A, Dworek N, Kasprzyk K, et al. Predictors of depressive symptoms in patients with stroke - a three-month follow-up. Neurol Neurochir Pol. 2010;44(1):13-20.
20. Hackett ML, Hill KM, Hewison J, Anderson CS, House AO. Stroke Survivors Who Score below Threshold on Standard Depression Measures May Still Have Negative Cognitions of Concern. Stroke. 2010;41(3):478-81.
21. Brown C, Hasson H, Thyselius V, Almborg AH. Post-stroke depression and functional independence: A conundrum. Acta Neurologica Scandinavica. 2012;126(1):45-51.
22.White JH, Attia J, Sturm J, Carter G, Magin P. Predictors of depression and anxiety in community dwelling stroke survivors: a cohort study. Disability and rehabilitation. 2014;36(23):1975-82.
23. De Ryck A, Fransen E, Brouns R, Geurden M, Peij D, Marien P, et al. Poststroke depression and its multifactorial nature: Results from a prospective longitudinal study. Journal of the Neurological Sciences. 2014;347(1-2):159-66.
24. Wichowicz HM, Gasecki D, Lass P, Landowski J, Swierkocka M, Wisniewski G, et al. Clinical utility of chosen factors in predicting post-stroke depression: a one year follow-up. Psychiatr Pol. 2015;49(4):683-96.

25. De Man-Van Ginkel JM, Hafsteinsdottir TB, Lindeman E, Geerlings MI, Grobbee DE, Schuurmans MJ. Clinical Manifestation of Depression after Stroke: Is It Different from Depression in Other Patient Populations? PLoS ONE. 2015;10(12).
26. Yu S, Arima H, Bertmar C, Hirakawa Y, Priglinger M, Evans K, et al. Depression but not anxiety predicts recurrent cerebrovascular events. Acta Neurologica Scandinavica. 2016;134(1):29-34.
27. Mutai H, Furukawa T, Houri A, Suzuki A, Hanihara T. Factors associated with multidimensional aspect of post-stroke fatigue in acute stroke period. Asian Journal of Psychiatry. 2017;26:1-5.
28. Barra M, Evensen GSH, Valeberg BT. Cues and clues predicting presence of symptoms of depression in stroke survivors. J Clin Nurs. 2017;26(3-4):546-56.
29. Stern-Nezer S, Eyngorn I, Mlynash M, Snider RW, Venkatsubramanian C, Wijman CAC, et al. Depression one year after hemorrhagic stroke is associated with late worsening of outcomes. Neurorehabilitation. 2017;41(1):179-87.
30. Fournier LE, Beauchamp JES, Zhang X, Bonojo E, Love M, Cooksey G, et al. Assessment of the Progression of Poststroke Depression in Ischemic Stroke Patients Using the Patient Health Questionnaire-9. Journal of Stroke & Cerebrovascular Diseases. 2020;29(4):8.
31. Ojagbemi A, Akinyemi J, Wahab K, Owolabi L, Arulogun O, Akpalu J, et al. Pre-Stroke Depression in Ghana and Nigeria: Prevalence, Predictors and Association With Poststroke Depression. Journal of Geriatric Psychiatry and Neurology. 2022;35(1):121-7.
32. Noushad N, Sachita S, Varughese SA, Joy SK, Jose S. Post stroke depression and anxiety: Prevalance and correlates. Asian Journal of Pharmaceutical and Clinical Research. 2021;14(9):142-7.
33. Al Qawasmeh M, Aldabbour B, Abuabada A, Abdelrahman K, Elamassie S, Khweileh M, et al. Prevalence, Severity, and Predictors of Poststroke Depression in a Prospective Cohort of Jordanian Patients. Stroke Research and Treatment. 2022;2022:6506326.
34. Ytterberg C, Cegrell L, von Koch L, Wiklander M. Depression symptoms 6 years after stroke are associated with higher perceived impact of stroke, limitations in ADL and restricted participation. Scientific Reports. 2022;12(1).
35. Sagen-Vik U, Finset A, Moum T, Vik TG, Dammen T. The longitudinal course of anxiety, depression and apathy through two years after stroke. Journal of Psychosomatic Research. 2022;162.

36. Ladwig S, Ziegler M, Sudmeyer M, Werheid K. The Post-Stroke Depression Risk Scale (PoStDeRiS): Development of an Acute-Phase Prediction Model for Depression 6 Months After Stroke. Journal of the Academy of Consultation-Liaison Psychiatry. 2022;63(2):144-52.
37. Wade DT, Legh-Smith J, Hewer RA. Depressed mood after stroke. A community study of its frequency. British Journal of Psychiatry. 1987;151(AUG.):200-5.
38. House A, Dennis M, Mogridge L, Warlow C, Hawton K, Jones L. Mood disorders in the year after first stroke. British Journal of Psychiatry. 1991;158(JAN.):83-92.
39. Burvill PW, Johnson GA, Jamrozik KD, Anderson CS, Stewart-Wynne EG, Chakera TMH. Prevalence of depression after stroke: The Perth Community Stroke Study. British Journal of Psychiatry. 1995;166(MAR.):320-7.
40. Kotila M, Numminen H, Waltimo O, Kaste M. Depression after stroke: Results of the FINNSTROKE study. Stroke. 1998;29(2):368-72.
41. Appelros P, Viitanen M. Prevalence and predictors of depression at one year in a Swedish population-based cohort with first-ever stroke. Journal of Stroke and Cerebrovascular Diseases. 2004;13(2):52-7.
42. Jonsson AC, Lindgren I, Hallstrom B, Norrving B, Lindgren A. Determinants of quality of life in stroke survivors and their informal caregivers. Stroke. 2005;36(4):803-8.
43. Paul SL, Dewey HM, Sturm JW, Macdonell RAL, Thrift AG. Prevalence of depression and use of antidepressant medication at 5-years poststroke in the North East Melbourne stroke incidence study. Stroke. 2006;37(11):2854-5.
44. Skaner Y, Nilsson GH, Sundquist K, Hassler E, Krakau I. Self-rated health, symptoms of depression and general symptoms at 3 and 12 months after a first-ever stroke: A municipality-based study in Sweden. BMC Family Practice. 2007;8.
45. Chausson N, Olindo S, Cabre P, Saint-Vil M, Smadja D. Five-year outcome of a stroke cohort in martinique, French West Indies: Etude realisee en martinique et centree sur l'incidence des accidents vasculaires cerebraux, part 2. Stroke. 2010;41(4):594-9.
46. Feigin VL, Barker-Collo S, Parag V, Senior H, Lawes CMM, Ratnasabapathy Y, et al. Auckland Stroke Outcomes Study: Part 1: Gender, stroke types, ethnicity, and functional outcomes 5 years poststroke. Neurology. 2010;75(18):1597-607.
47. Liman TG, Heuschmann PU, Endres M, Floel A, Schwab S, Kolominsky-Rabas PL. Impact of low mini-mental status on health outcome up to 5 years after stroke: the Erlangen Stroke Project. J Neurol. 2012;259(6):1125-30.

48. Zhang N, Wang CX, Wang AX, Bai Y, Zhou Y, Wang YL, et al. Time course of depression and one-year prognosis of patients with stroke in mainland China. CNS Neuroscience & Therapeutics. 2012;18(6):475-81.
49. Ayerbe L, Ayis S, Crichton S, Wolfe CDA, Rudd AG. The natural history of depression up to 15 years after stroke: The South London stroke register. Stroke. 2013;44(4):1105-10.
50. McCarthy MJ, Sucharew HJ, Alwell K, Moomaw CJ, Woo D, Flaherty ML, et al. Age, subjective stress, and depression after ischemic stroke. Journal of behavioral medicine. 2016;39(1):55-64.
51. Arba F, Ali M, Quinn TJ, Hankey GJ, Lees KR, Inzitari D. Lacunar Infarcts, Depression, and Anxiety Symptoms One Year after Stroke. Journal of Stroke and Cerebrovascular Diseases. 2016;25(4):831-4.
52. El Husseini N, Goldstein LB, Peterson ED, Zhao X, Olson DM, Williams JW, Jr., et al. Depression Status Is Associated with Functional Decline Over 1-Year Following Acute Stroke. Journal of Stroke & Cerebrovascular Diseases. 2017;26(7):1393-9.
53. Barker-Collo S, Krishnamurthi R, Witt E, Theadom A, Starkey N, Barber PA, et al. Depression and Anxiety Across the First Year After Ischemic Stroke: Findings from a Population-Based New Zealand ARCOS-IV Study. Brain Impair. 2017;18(3):265-76
54. Bovim MR, Lndredavik B, Hokstad A, Cumming T, Bernhardt J, Askim T. Relationship between pre-stroke physical activity and symptoms of post-stroke anxiety and depression. J Rehabil Med. 2019;51(10):755-60.
55. Kowalska K, Dros J, Mazurek M, Pasinska P, Gorzkowska A, Klimkowicz-Mrowiec A. Delirium post-stroke: Short-and long-term effect on depression, anxiety, apathy and aggression (research study-part of propolis study). Journal of Clinical Medicine. 2020;9(7):1-11
56. Dong L, Sanchez BN, Skolarus LE, Stulberg E, Morgenstern LB, Lisabeth LD. Sex difference in prevalence of depression after stroke. Neurology. 2020;94(19):e1973-e83.
57. Roth DL, Haley WE, Sheehan OC, Liu C, Clay OJ, Rhodes JD, et al. Depressive Symptoms After Ischemic Stroke Population-Based Comparisons of Patients and Caregivers With Matched Controls. Stroke. 2020;51(1):54-60.
58. Williams OA, Demeyere N. Association of Depression and Anxiety With Cognitive Impairment 6 Months After Stroke. Neurology. 2021;96(15):E1966-E74.

59. Ojagbemi A, Akinyemi J, Wahab K, Owolabi L, Arulogun O, Akpalu J, et al. Pre-Stroke Depression in Ghana and Nigeria: Prevalence, Predictors and Association With Poststroke Depression. Journal of Geriatric Psychiatry and Neurology. 2022;35(1):121-7.
60. Shin M, Sohn MK, Lee J, Kim DY, Shin YI, Oh GJ, et al. Post-Stroke Depression and Cognitive Aging: A Multicenter, Prospective Cohort Study. Journal of Personalized Medicine. 2022;12(3).
61. Blochl M, Nestler S. Long-term Changes in Depressive Symptoms before and after Stroke. Neurology. 2022;99(7):E720-E9.
62. Carod-Artal J, Egido JA, Gonzalez JL, de Seijas EV. Quality of life among stroke survivors evaluated 1 year after stroke - Experience of a stroke unit. Stroke. 2000;31(12):2995-3000.
63. Gillen R, Tennen H, McKee TE, Gernert-Dott P, Affleck G. Depressive symptoms and history of depression predict rehabilitation efficiency in stroke patients. Arch Phys Med Rehabil. 2001;82(12):1645-9.
64. Tang WK, Ungvari GS, Chiu HFK, Sze KH, Woo J, Kay R. Psychiatric morbidity in first time stroke patients in Hong Kong: A pilot study in a rehabilitation unit. Australian and New Zealand Journal of Psychiatry. 2002;36(4):544-9.
65. Barker-Collo SL. Depression and anxiety 3 months post stroke: Prevalence and correlates. Archives of Clinical Neuropsychology. 2007;22(4):519-31.
66. Saxena SK, Ng TP, Yong D, Fong NP, Koh G. Subthreshold depression and cognitive impairment but not demented in stroke patients during their rehabilitation. Acta Neurologica Scandinavica. 2008;117(2):133-40.
67.Townend BS, Whyte S, Desborough T, Crimmins D, Markus R, Levi C, et al. Longitudinal prevalence and determinants of early mood disorder post-stroke. J Clin Neurosci. 2007;14(5):429-34.
68. Paolucci S, Di Vita A, Massicci R, Traballesi M, Bureca I, Matano A, et al. Impact of participation on rehabilitation Results: A multivariate study. European Journal of Physical and Rehabilitation Medicine. 2012;48(3):455-66.
69. Mutai H, Furukawa T, Araki K, Misawa K, Hanihara T. Long-term outcome in stroke survivors after discharge from a convalescent rehabilitation ward. Psychiatry and Clinical Neurosciences. 2013;67(6):434-40.
70. Abdul-sattar AB, Godab T. Predictors of functional outcome in Saudi Arabian patients with stroke after inpatient rehabilitation. Neurorehabilitation. 2013;33(2):209-16.

71. Schottke H, Giabbiconi CM. Post-stroke depression and post-stroke anxiety: Prevalence and predictors. International Psychogeriatrics. 2015;27(11):1805-12.
72. Ng YS, Tan KH, Chen C, Senolos GC, Koh GC. How Do Recurrent and First-Ever Strokes Differ in Rehabilitation Outcomes? American journal of physical medicine & rehabilitation. 2016;95(10):709-17.
73. Liman TG, Heuschmann PU, Endres M, Floel A, Schwab S, Kolominsky-Rabas PL. Impact of low mini-mental status on health outcome up to 5 years after stroke: the Erlangen Stroke Project. J Neurol. 2012;259(6):1125-30.
74. Broussy S, Saillour-Glenisson F, Garcia-Lorenzo B, Rouanet F, Lesaine E, Maugeais M, et al. Sequelae and quality of life in patients living at home 1 year after a stroke managed in stroke units. Frontiers in Neurology. 2019;10(AUG).
75. Volz M, Ladwig S, Werheid K. Gender differences in post-stroke depression: A longitudinal analysis of prevalence, persistence and predictive value of known risk factors. Neuropsychological rehabilitation. 2021;31(1):1-17.
76. Suzuki, A., et al. The Prevalence and Course of Neuropsychiatric Symptoms in Stroke Patients Impact Functional Recovery During in-Hospital Rehabilitation.Topics in Stroke Rehabilitation 29(1) (2022): 1-8.
77. Stokman-Meiland DCM, Groeneveld IF, Arwert HJ, van der Pas SL, Meesters JJL, Mishre RRD, et al. The course of depressive symptoms in the first 12 months post-stroke and its association with unmet needs. Disability and Rehabilitation. 2022;44(3):428-35.
