# Supplementary material for: Prevalence and natural history of depression after stroke: A systematic review and meta-analysis of observational studies
Source: PLoS Med. 2023 Mar 28;20(3):e1004200. doi: 10.1371/journal.pmed.1004200 (PMC10047522; doi:10.1371/journal.pmed.1004200)
Supplement: S4 Fig — (PDF) [file pmed.1004200.s010.pdf]

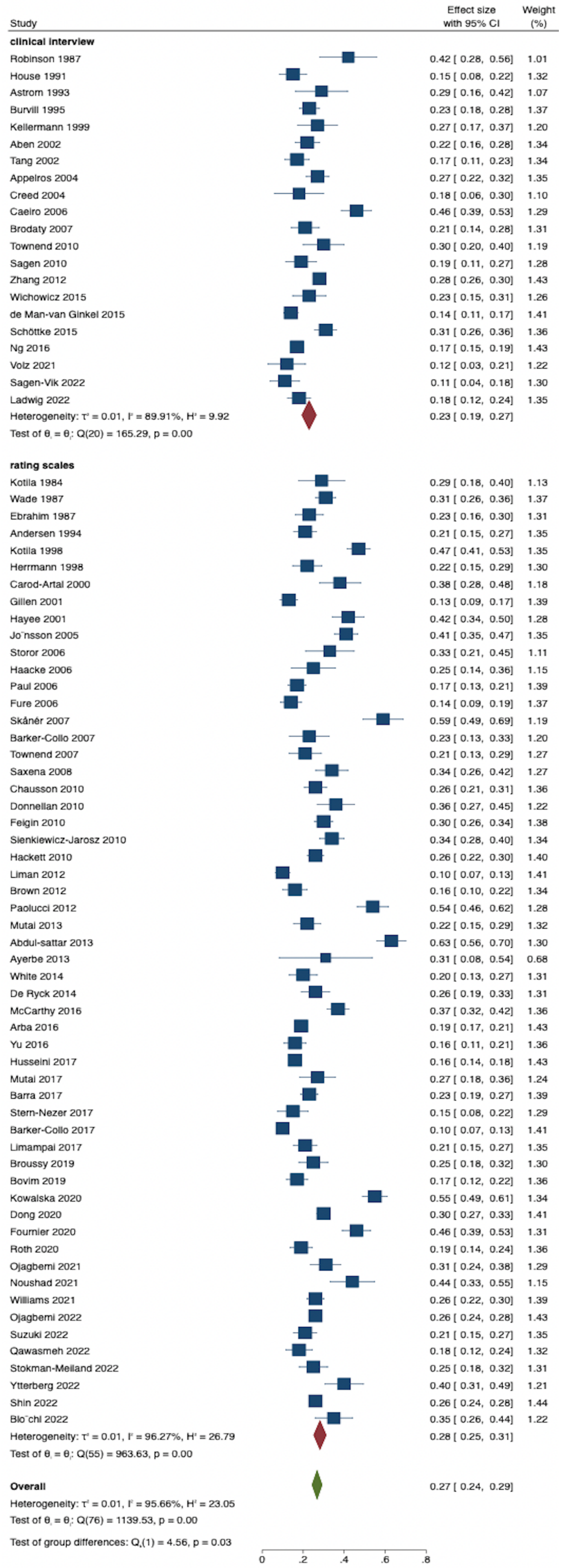

Random-effects REML model

Reference

1.Robinson RG, Bolduc PL, Price TR. Two-year longitudinal study of poststroke mood disorders: Diagnosis and outcome at one and two years. Stroke. 1987;18(5):837-43.

2.House A, Dennis M, Mogridge L, Warlow C, Hawton K, Jones L. Mood disorders in the year after first stroke. British Journal of Psychiatry. 1991;158(JAN.):83-92.

3.Astrom M, Adolfsson R, Asplund K. Major depression in stroke patients: A 3-year longitudinal study. Stroke. 1993;24(7):976-82.

4.Burvill PW, Johnson GA, Jamrozik KD, Anderson CS, Stewart-Wynne EG, Chakera TMH. Prevalence of depression after stroke: The Perth Community Stroke Study. British Journal of Psychiatry. 1995;166(MAR.):320-7.

5.Kellermann M, Fekete I, Gesztelyi R, Csiba L, Kollar J, Sikula J, et al. Screening for depressive symptoms in the acute phase of stroke. General Hospital Psychiatry. 1999;21(2):116-21.

6. Aben I, Verhey F, Lousberg R, et al. Validity of the Beck depression inventory, hospital anxiety and depression scale, SCL-90, and Hamilton depression rating scale as screening instruments for depression in stroke patients. Psychosomatics 2002;43:386–93.

7. Tang WK, Ungvari GS, Chiu HFK, Sze KH, Woo J, Kay R. Psychiatric morbidity in first time stroke patients in Hong Kong: A pilot study in a rehabilitation unit. Australian and New Zealand Journal of Psychiatry. 2002;36(4):544-9.

8. Appelros P, Viitanen M. Prevalence and predictors of depression at one year in a Swedish population-based cohort with first-ever stroke. Journal of Stroke and Cerebrovascular Diseases. 2004;13(2):52-7.

9. Creed A, Swanwick G, O'Neill D. Screening for post stroke depression in patients with acute stroke including those with communication disorders. International Journal of Geriatric Psychiatry. 2004;19(6):595-7.

10.Caeiro L, Ferro JM, Santos CO, Figueira ML. Depression in acute stroke. Journal of Psychiatry and Neuroscience. 2006;31(6):377-83.

11. Brodaty H, Withall A, Altendorf A, Sachdev PS. Rates of depression at 3 and 15 months poststroke and their relationship with cognitive decline: The Sydney stroke study. American Journal of Geriatric Psychiatry. 2007;15(6):477-86.

12.Townend E, Tinson D, Kwan J, Sharpe M. 'Feeling sad and useless': an investigation into personal acceptance of disability and its association with depression following stroke. Clin Rehabil. 2010;24(6):555-64.
13.Sagen-Vik U, Finset A, Moum T, Vik TG, Dammen T. The longitudinal course of anxiety, depression and apathy through two years after stroke. Journal of Psychosomatic Research. 2022;162.
14.Zhang N, Wang CX, Wang AX, Bai Y, Zhou Y, Wang YL, et al. Time course of depression and one-year prognosis of patients with stroke in mainland China. CNS Neuroscience & Therapeutics. 2012;18(6):475-81.
15.Wichowicz HM, Gasecki D, Lass P, Landowski J, Swierkocka M, Wisniewski G, et al. Clinical utility of chosen factors in predicting post-stroke depression: a one year follow-up. Psychiatr Pol. 2015;49(4):683-96.
16.De Man-Van Ginkel JM, Hafsteinsdottir TB, Lindeman E, Geerlings MI, Grobbee DE, Schuurmans MJ. Clinical Manifestation of Depression after Stroke: Is It Different from Depression in Other Patient Populations? PLoS ONE. 2015;10(12).
17.Schottke H, Giabbiconi CM. Post-stroke depression and post-stroke anxiety: Prevalence and predictors. International Psychogeriatrics. 2015;27(11):1805-12.
18.Ng YS, Tan KH, Chen C, Senolos GC, Koh GC. How Do Recurrent and First-Ever Strokes Differ in Rehabilitation Outcomes? American journal of physical medicine & rehabilitation. 2016;95(10):709-17.
19.Volz M, Ladwig S, Werheid K. Gender differences in post-stroke depression: A longitudinal analysis of prevalence, persistence and predictive value of known risk factors. Neuropsychological rehabilitation. 2021;31(1):1-17.
20.Sagen-Vik U, Finset A, Moum T, Vik TG, Dammen T. The longitudinal course of anxiety, depression and apathy through two years after stroke. Journal of Psychosomatic Research. 2022;162.
21.Ladwig S, Ziegler M, Sudmeyer M, Werheid K. The Post-Stroke Depression Risk Scale (PoStDeRiS): Development of an Acute-Phase Prediction Model for Depression 6 Months After Stroke. Journal of the Academy of Consultation-Liaison Psychiatry. 2022;63(2):144-52.
22.Kotila M, Waltimo O, Niemi ML, Laaksonen R, Lempinen M. The profile of recovery from stroke and factors influencing outcome. Stroke. 1984;15(6):1039-44.

23.Wade DT, Legh-Smith J, Hewer RA. Depressed mood after stroke. A community study of its frequency. British Journal of Psychiatry. 1987;151(AUG.):200-5.
24.Ebrahim S, Barer D, Nouri F. Affective illness after stroke. British Journal of Psychiatry. 1987;151:52-6.
25.Andersen G, Vestergaard K, Riis JO, Lauritzen L. Incidence of post-stroke depression during the first year in a large unselected stroke population determined using a valid standardized rating scale. Acta Psychiatrica Scandinavica. 1994;90(3):190-5.
26.Kotila M, Numminen H, Waltimo O, Kaste M. Depression after stroke: Results of the FINNSTROKE study. Stroke. 1998;29(2):368-72.
27.Herrmann N, Black SE, Lawrence J, Szekely C, Szalai JP. The Sunnybrook stroke study - A prospective study of depressive symptoms and functional outcome. Stroke. 1998;29(3):618-24.
28.Carod-Artal J, Egido JA, Gonzalez JL, de Seijas EV. Quality of life among stroke survivors evaluated 1 year after stroke - Experience of a stroke unit. Stroke. 2000;31(12):2995-3000.
29.Gillen R, Tennen H, McKee TE, Gernert-Dott P, Affleck G. Depressive symptoms and history of depression predict rehabilitation efficiency in stroke patients. Arch Phys Med Rehabil. 2001;82(12):1645-9.
30.Hayee MA, Akhtar N, Haque A, Rabbani MG. Depression after stroke-analysis of 297 stroke patients. Bangladesh Medical Research Council Bulletin. 2001;27(3):96-102.
31.Jonsson AC, Lindgren I, Hallstrom B, Norrving B, Lindgren A. Determinants of quality of life in stroke survivors and their informal caregivers. Stroke. 2005;36(4):803-8.
32.Storor DL, Byrne GJ. Pre-morbid personality and depression following stroke. International Psychogeriatrics. 2006;18(3):457-69.
33.Haacke C, Althaus A, Spottke A, Siebert U, Back T, Dodel R. Long-term outcome after stroke - Evaluating health-related quality of life using utility measurements. Stroke. 2006;37(1):193-8.
34.Paul SL, Dewey HM, Sturm JW, Macdonell RAL, Thrift AG. Prevalence of depression and use of antidepressant medication at 5-years poststroke in the North East Melbourne stroke incidence study. Stroke. 2006;37(11):2854-5.
35.Fure B, Wyller TB, Engedal K, Thommessen B. Emotional symptoms in acute ischemic stroke. International Journal of Geriatric Psychiatry. 2006;21(4):382-7.

36.Skaner Y, Nilsson GH, Sundquist K, Hassler E, Krakau I. Self-rated health, symptoms of depression and general symptoms at 3 and 12 months after a first-ever stroke: A municipality-based study in Sweden. BMC Family Practice. 2007;8.
37.Barker-Collo SL. Depression and anxiety 3 months post stroke: Prevalence and correlates. Archives of Clinical Neuropsychology. 2007;22(4):519-31.
38.Townend BS, Whyte S, Desborough T, Crimmins D, Markus R, Levi C, et al. Longitudinal prevalence and determinants of early mood disorder post-stroke. J Clin Neurosci. 2007;14(5):429-34.
39.Saxena SK, Ng TP, Yong D, Fong NP, Koh G. Subthreshold depression and cognitive impairment but not demented in stroke patients during their rehabilitation. Acta Neurologica Scandinavica. 2008;117(2):133-40.
40.Chausson N, Olindo S, Cabre P, Saint-Vil M, Smadja D. Five-year outcome of a stroke cohort in martinique, French West Indies: Etude realisee en martinique et centree sur l'incidence des accidents vasculaires cerebraux, part 2. Stroke. 2010;41(4):594-9.
41.Donnellan C, Hickey A, Hevey D, O'Neill D. Effect of mood symptoms on recovery one year after stroke. International Journal of Geriatric Psychiatry. 2010;25(12):1288-95.
42.Feigin VL, Barker-Collo S, Parag V, Senior H, Lawes CMM, Ratnasabapathy Y, et al. Auckland Stroke Outcomes Study: Part 1: Gender, stroke types, ethnicity, and functional outcomes 5 years poststroke. Neurology. 2010;75(18):1597-607.
43.Sienkiewicz-Jarosz H, Milewska D, Bochynska A, Chelmniak A, Dworek N, Kasprzyk K, et al. Predictors of depressive symptoms in patients with stroke - a three-month follow-up. Neurol Neurochir Pol. 2010;44(1):13-20.
44.Hackett ML, Hill KM, Hewison J, Anderson CS, House AO. Stroke Survivors Who Score below Threshold on Standard Depression Measures May Still Have Negative Cognitions of Concern. Stroke. 2010;41(3):478-81.
45.Liman TG, Heuschmann PU, Endres M, Floel A, Schwab S, Kolominsky-Rabas PL. Impact of low mini-mental status on health outcome up to 5 years after stroke: the Erlangen Stroke Project. J Neurol. 2012;259(6):1125-30.
46.Brown C, Hasson H, Thyselius V, Almborg AH. Post-stroke depression and functional independence: A conundrum. Acta Neurologica Scandinavica. 2012;126(1):45-51.

47.Paolucci S, Di Vita A, Massicci R, Traballesi M, Bureca I, Matano A, et al. Impact of participation on rehabilitation Results: A multivariate study. European Journal of Physical and Rehabilitation Medicine. 2012;48(3):455-66.
48.Mutai H, Furukawa T, Araki K, Misawa K, Hanihara T. Long-term outcome in stroke survivors after discharge from a convalescent rehabilitation ward. Psychiatry and Clinical Neurosciences. 2013;67(6):434-40.
49.Abdul-sattar AB, Godab T. Predictors of functional outcome in Saudi Arabian patients with stroke after inpatient rehabilitation. Neurorehabilitation. 2013;33(2):209-16.
50. Ayerbe L, Ayis S, Crichton S, Wolfe CDA, Rudd AG. The natural history of depression up to 15 years after stroke: The South London stroke register. Stroke. 2013;44(4):1105-10.
51.White JH, Attia J, Sturm J, Carter G, Magin P. Predictors of depression and anxiety in community dwelling stroke survivors: a cohort study. Disability and rehabilitation. 2014;36(23):1975-82.
52.De Ryck A, Fransen E, Brouns R, Geurden M, Peij D, Marien P, et al. Poststroke depression and its multifactorial nature: Results from a prospective longitudinal study. Journal of the Neurological Sciences. 2014;347(1-2):159-66.
53.McCarthy MJ, Sucharew HJ, Alwell K, Moomaw CJ, Woo D, Flaherty ML, et al. Age, subjective stress, and depression after ischemic stroke. Journal of behavioral medicine. 2016;39(1):55-64.
54.Arba F, Ali M, Quinn TJ, Hankey GJ, Lees KR, Inzitari D. Lacunar Infarcts, Depression, and Anxiety Symptoms One Year after Stroke. Journal of Stroke and Cerebrovascular Diseases. 2016;25(4):831-4.
55.Yu S, Arima H, Bertmar C, Hirakawa Y, Priglinger M, Evans K, et al. Depression but not anxiety predicts recurrent cerebrovascular events. Acta Neurologica Scandinavica. 2016;134(1):29-34.
56.El Husseini N, Goldstein LB, Peterson ED, Zhao X, Olson DM, Williams JW, Jr., et al. Depression Status Is Associated with Functional Decline Over 1-Year Following Acute Stroke. Journal of Stroke & Cerebrovascular Diseases. 2017;26(7):1393-9.
57.Mutai H, Furukawa T, Houri A, Suzuki A, Hanihara T. Factors associated with multidimensional aspect of post-stroke fatigue in acute stroke period. Asian Journal of Psychiatry. 2017;26:1-5.

58.Barra M, Evensen GSH, Valeberg BT. Cues and clues predicting presence of symptoms of depression in stroke survivors. J Clin Nurs. 2017;26(3-4):546-56.
59.Stern-Nezer S, Eyngorn I, Mlynash M, Snider RW, Venkatsubramanian C, Wijman CAC, et al. Depression one year after hemorrhagic stroke is associated with late worsening of outcomes. Neurorehabilitation. 2017;41(1):179-87.
60.Barker-Collo S, Krishnamurthi R, Witt E, Theadom A, Starkey N, Barber PA, et al. Depression and Anxiety Across the First Year After Ischemic Stroke: Findings from a Population-Based New Zealand ARCOS-IV Study. Brain Impair. 2017;18(3):265-76
61.Limampai P, Wongsrithep W, Kuptniratsaikul V. Depression after stroke at 12-month follow-up: a multicenter study. International Journal of Neuroscience. 2017;127(10):887-92.
62.Broussy S, Saillour-Glenisson F, Garcia-Lorenzo B, Rouanet F, Lesaine E, Maugeais M, et al. Sequelae and quality of life in patients living at home 1 year after a stroke managed in stroke units. Frontiers in Neurology. 2019;10(AUG).
63.Bovim MR, Lndredavik B, Hokstad A, Cumming T, Bernhardt J, Askim T. Relationship between pre-stroke physical activity and symptoms of post-stroke anxiety and depression. J Rehabil Med. 2019;51(10):755-60.
64.Kowalska K, Dros J, Mazurek M, Pasinska P, Gorzkowska A, Klimkowicz-Mrowiec A. Delirium post-stroke: Short-and long-term effect on depression, anxiety, apathy and aggression (research study-part of propolis study). Journal of Clinical Medicine. 2020;9(7):1-11
65.Dong L, Sanchez BN, Skolarus LE, Stulberg E, Morgenstern LB, Lisabeth LD. Sex difference in prevalence of depression after stroke. Neurology. 2020;94(19):e1973-e83.
66.Fournier LE, Beauchamp JES, Zhang X, Bonojo E, Love M, Cooksey G, et al. Assessment of the Progression of Poststroke Depression in Ischemic Stroke Patients Using the Patient Health Questionnaire-9. Journal of Stroke & Cerebrovascular Diseases. 2020;29(4):8.
67.Roth DL, Haley WE, Sheehan OC, Liu C, Clay OJ, Rhodes JD, et al. Depressive Symptoms After Ischemic Stroke Population-Based Comparisons of Patients and Caregivers With Matched Controls. Stroke. 2020;51(1):54-60.
68.Ojagbemi A, Bello T, Owolabi M, Baiyewu O. Prevalence, predictors, and prognoses of prestroke neuropsychiatric symptoms at 3 months poststroke. International Psychogeriatrics. 2021;33(8):827-34.

69.Noushad N, Sachita S, Varughese SA, Joy SK, Jose S. Post stroke depression and anxiety: Prevalance and correlates. Asian Journal of Pharmaceutical and Clinical Research. 2021;14(9):142-7.
70.Williams OA, Demeyere N. Association of Depression and Anxiety With Cognitive Impairment 6 Months After Stroke. Neurology. 2021;96(15):E1966-E74.
71.Ojagbemi A, Akinyemi J, Wahab K, Owolabi L, Arulogun O, Akpalu J, et al. Pre-Stroke Depression in Ghana and Nigeria: Prevalence, Predictors and Association With Poststroke Depression. Journal of Geriatric Psychiatry and Neurology. 2022;35(1):121-7.
72.Suzuki, A., et al. The Prevalence and Course of Neuropsychiatric Symptoms in Stroke Patients Impact Functional Recovery During in-Hospital Rehabilitation.Topics in Stroke Rehabilitation 29(1) (2022): 1-8.
73.Al Qawasmeh M, Aldabbour B, Abuabada A, Abdelrahman K, Elamassie S, Khweileh M, et al. Prevalence, Severity, and Predictors of Poststroke Depression in a Prospective Cohort of Jordanian Patients. Stroke Research and Treatment. 2022;2022:6506326.
74.Stokman-Meiland DCM, Groeneveld IF, Arwert HJ, van der Pas SL, Meesters JJL, Mishre RRD, et al. The course of depressive symptoms in the first 12 months post-stroke and its association with unmet needs. Disability and Rehabilitation. 2022;44(3):428-35.
75.Ytterberg C, Cegrell L, von Koch L, Wiklander M. Depression symptoms 6 years after stroke are associated with higher perceived impact of stroke, limitations in ADL and restricted participation. Scientific Reports. 2022;12(1).
76.Shin M, Sohn MK, Lee J, Kim DY, Shin YI, Oh GJ, et al. Post-Stroke Depression and Cognitive Aging: A Multicenter, Prospective Cohort Study. Journal of Personalized Medicine. 2022;12(3).
77.Blochl M, Nestler S. Long-term Changes in Depressive Symptoms before and after Stroke. Neurology. 2022;99(7):E720-E9.
