# Supplementary material for: Prevalence and natural history of depression after stroke: A systematic review and meta-analysis of observational studies
Source: PLoS Med. 2023 Mar 28;20(3):e1004200. doi: 10.1371/journal.pmed.1004200 (PMC10047522; doi:10.1371/journal.pmed.1004200)
Supplement: S10 Fig — (PDF) [file pmed.1004200.s016.pdf]

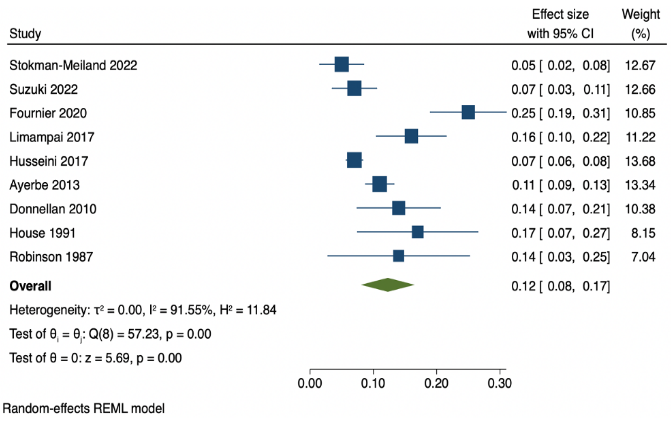

Reference

1.Stokman-Meiland DCM, Groeneveld IF, Arwert HJ, van der Pas SL, Meesters JJL, Mishre RRD, et al. The course of depressive symptoms in the first 12 months post-stroke and its association with unmet needs. Disability and Rehabilitation. 2022;44(3):428-35.

2.Suzuki, A., et al. The Prevalence and Course of Neuropsychiatric Symptoms in Stroke Patients Impact Functional Recovery During in-Hospital Rehabilitation.Topics in Stroke Rehabilitation 29(1) (2022): 1-8.

3.Fournier LE, Beauchamp JES, Zhang X, Bonojo E, Love M, Cooksey G, et al. Assessment of the Progression of Poststroke Depression in Ischemic Stroke Patients Using the Patient Health Questionnaire-9. Journal of Stroke & Cerebrovascular Diseases. 2020;29(4):8.

4.Limampai P, Wongsrithep W, Kuptniratsaikul V. Depression after stroke at 12-month follow-up: a multicenter study. International Journal of Neuroscience. 2017;127(10):887-92.

5.El Husseini N, Goldstein LB, Peterson ED, Zhao X, Olson DM, Williams JW, Jr., et al. Depression Status Is Associated with Functional Decline Over 1-Year Following Acute Stroke. Journal of Stroke & Cerebrovascular Diseases. 2017;26(7):1393-9.

6. Ayerbe L, Ayis S, Crichton S, Wolfe CDA, Rudd AG. The natural history of depression up to 15 years after stroke: The South London stroke register. Stroke. 2013;44(4):1105-10.

7.Donnellan C, Hickey A, Hevey D, O'Neill D. Effect of mood symptoms on recovery one year after stroke. International Journal of Geriatric Psychiatry. 2010;25(12):1288-95.

8.House A, Dennis M, Mogridge L, Warlow C, Hawton K, Jones L. Mood disorders in the year after first stroke. British Journal of Psychiatry. 1991;158(JAN.):83-92.

9.Robinson RG, Bolduc PL, Price TR. Two-year longitudinal study of poststroke mood disorders: Diagnosis and outcome at one and two years. Stroke. 1987;18(5):837-43.
